# Overview of Some Second- and Third-Row Late Transition Metal Pincer-Type *N*-Heterocyclic Carbene Complexes: Synthesis, Optical Properties, and Applications

**DOI:** 10.3390/molecules30122640

**Published:** 2025-06-18

**Authors:** Dong-Ling Kuang, Ka-Kit Li, Lai-Hon Chung, Jun He, Chun-Yuen Wong

**Affiliations:** 1 School of Chemical Engineering and Light Industry, Guangdong University of Technology, Guangzhou 510006, China; 2 Department of Chemistry, City University of Hong Kong, Tat Chee Avenue, Kowloon, Hong Kong SAR, China; 3 Guangdong Provincial Laboratory of Chemistry and Fine Chemical Engineering Jieyang Center, Jieyang 515200, China

**Keywords:** *N*-heterocyclic carbene, coordination complexes, transition metal

## Abstract

*N*-heterocyclic carbenes (NHCs) were first isolated as stable species by Arduengo in 1991. Since then, they have expanded the boundaries of carbene chemistry and sparked extensive research. Utilizing NHCs to modify the electronic properties of transition metal complexes represents a significant advancement in the field. Pincer-type NHCs, which occupy half or more of the vacant sites on metal centers, typically result in structurally well-defined molecular platforms with specific active sites for a variety of applications. This review provides an overview of late transition metal complexes based on pincer-type NHCs, discussing their synthetic strategies, reactivities, and electronic properties, as well as their applications. Additionally, some perspectives will be presented to highlight future directions in this rapidly growing field.

## 1. Introduction

Sparked off by the isolation of stable *N*-heterocyclic carbenes (NHCs) by Arduengo [1], this category of organic species has been explored intensively and found thousands of applications. Over the past three decades, these ligands have become widely used in areas such as catalytic organic transformation [2,3], organic light-emitting diodes (OLEDs) [4], and metallodrugs [5,6]. NHCs are primarily known for their strong σ-donating ability, while their π-acceptance is generally limited [7], although exceptions exist where metal-NHC π-interactions play a role. These characteristic features make NHCs good partners with transition metal ions to give unlimited combinations of functional coordination compounds. Especially thanks to the strong σ-donating power of NHC, the potential for modulating the photoactiveness of coordination complexes is manifold when compared with classical polypyridyl ligands [8]. Utilizing NHCs to construct photoluminescent and photocatalytically active complexes is going to be mainstream for light-related applications.

**Figure 1:**
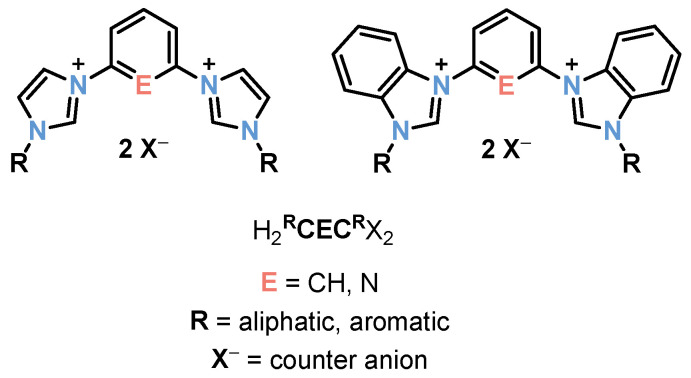
The general structure of pincer-type bis-NHC ligands to be discussed in this review.

Simple NHCs start with monodentate analogues, which are still labile when exposed to harsh conditions (i.e., strongly acidic conditions, high concentration of competitive ligands) and have limited functional tunability for modulating the electronic structure of the metal centers [9,10]. In contrast, multidentate (pincer-type) NHCs offer multichelation for stable molecular motifs and broader, tunable utility for harnessing the electronic structure of metal active sites, resulting in better application performance [11]. In earlier times, a milestone review on coordination compounds bearing pincer-type NHCs was reported by Danopoulos and co-workers [12]. Since then pincer-type NHC-based coordination complexes have captured more attention, and various reviews on this topic have been published during the last two decades [4,13,14]. This review aims to provide a focused overview of transition metal-NHC complexes based on the structural design of pincer ligands, which consist of two key components that securely anchor the ligand to the metal core. The first component is an aryl/pyridine ring directly σ-bonded to the metal, while the second consists of NHCs coordinating the metal center through the formation of M–C_NHC_ bonds. These NHCs associate with the central aryl/pyridine ring chelate metal centers to form two interconnected stable five-membered metallocycles. The modularity of these ligands provides precise control over the metal’s local environment, allowing for tuning through both steric and electronic effects. The classical structure of these ligands, known as “[H_2_**^R^CEC^R^**]^2+^” (R = aliphatic or aromatic groups; E = CH, N), is illustrated in Figure 1 [8].

This review begins with recent developments in the synthesis of M-^R^CEC^R^ complexes of late transition metal species including Ruthenium(II), Osmium(II), Rhodium(III), and Iridium(III). These complexes, with their *d*^6^ electronic configuration, typically exhibit octahedral coordination geometries, significant spin-orbit coupling, pronounced ligand field splitting, and strong coordination bonds [7]. Most of these M-NHC compounds show interesting photophysical or photoluminescent properties primarily based on triplet metal-to-ligand charge transfer (^3^MLCT), ligand-to-ligand charge transfer (^3^LLCT), or ligand-centered transition (^3^LC) excited states [8]. The discussion will then cover their fascinating photophysical properties and emerging applications across various fields.

## 2. Preparation and Reactivities of d^6^ Late Transition Metal-NHC Complexes

### 2.1. Ru/Os-^R^CNC^R^ Complex

**Scheme 1:**
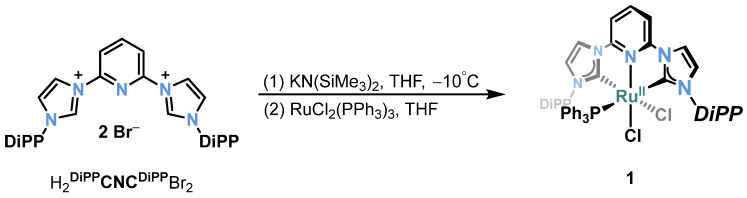
Synthetic route for Ru(II) bis-NHC complex **1**.

**Figure 2:**
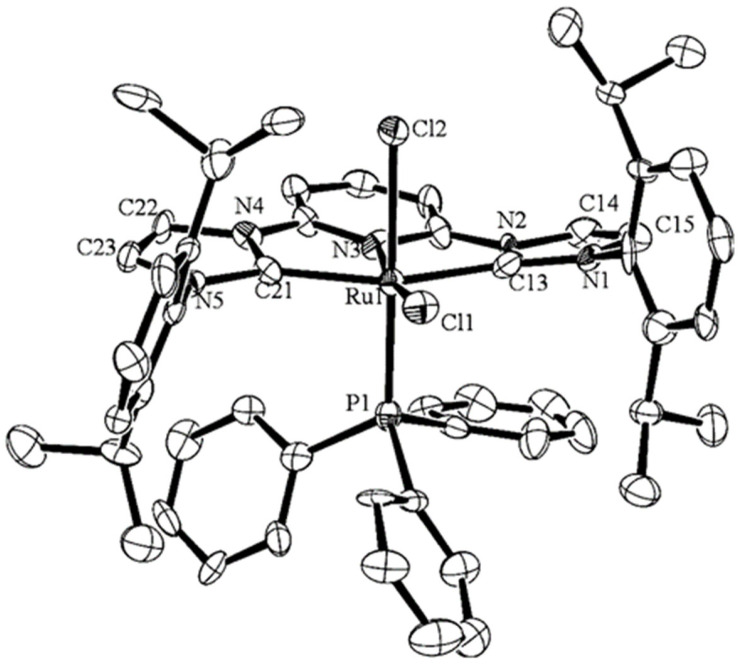
Molecular structure of **1**. Thermal ellipsoids show at 50% probability. The asymmetric unit contains two molecules and two molecules of dichloromethane. Selected bond lengths (Å): C(13)-Ru(1) 2.025(8), C(21)-Ru(1) 2.028(8), N(3)-Ru(1) 1.971(7), Cl(1)-Ru(1) 2.424(2), Cl(2)-Ru(1) 2.478(2), Ru(1)-P(1) 2.318(2).

In 2002, Danopoulos and co-workers reported the first Ru(II)-NHC complexes using biscarbene ligands, establishing a foundation for Ru-based complexes with multidentate NHC ligands. These complexes are versatile for catalytic applications. Notably, the **^DiPP^CNC^DiPP^** (DiPP = Diisopropylphenyl), a 2,6-[(*o*-dialkyl)phenylimidazolylidene]pyridine, was synthesized by reacting 2,6-[(*o*-dialkyl)phenylimidazolium]pyridine dibromide with excess KN(SiMe_3_)_2_ in THF at –10 °C. The resulting **^DiPP^CNC^DiPP^** ligand was allowed to react with RuCl_2_(PPh_3_)_3_ in THF to yield the air-stable orange X-ray crystallographically resolved Ru(II) bis-NHC complex **1** (Scheme 1 and Figure 2) [15].

**Scheme 2:**
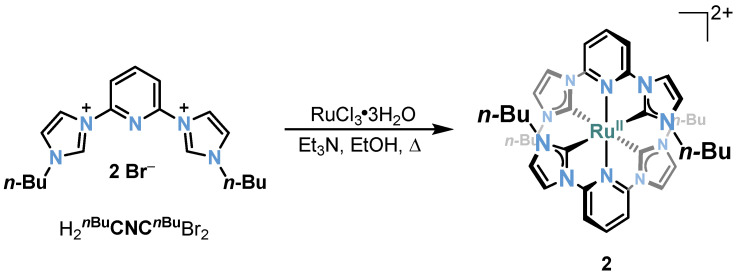
Synthetic route for **[Ru^II^(*^n^*^Bu^CNC*^n^*^Bu^)_2_]^2+^**, **2**.

In 2003, Poyatos and co-workers successfully synthesized **[Ru^II^(*^n^*^Bu^CNC*^n^*^Bu^)_2_]^2+^** (**2**) in a modest yield (17%). The synthesis involved reacting RuCl_3_•3H_2_O with H_2_***^n^*^Bu^CNC*^n^*^Bu^**Br_2_, 2,6-[*n*-butylimidazolium]pyridine dibromide, and NEt_3_ in refluxing EtOH, followed by counterion exchange and column chromatography (Scheme 2). The resulting complex is bright yellow and air-stable. X-ray crystallography revealed that the two CNC pincers coordinate the Ru center in a meridional mode, with the constituent rings nearly coplanar. The Ru center adopts a distorted octahedral geometry, with the *n*-butyl groups extending out of the coordination plane. Ru–C bond lengths within the imidazolyl moiety were measured at 2.05–2.07 Å, indicating a predominantly single-bond character with minimal backdonation [16].

**Scheme 3:**
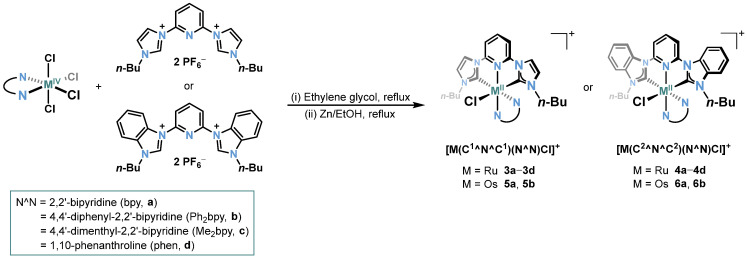
Synthetic routes for **[Ru^II^/Os^II^(C^N^C)(N^N)Cl]^+^**.

**Figure 3:**
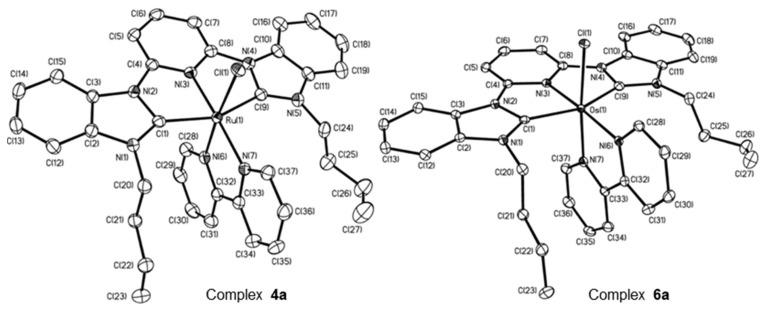
Perspective views of **4a**, and **6a** as represented by 30% probability ellipsoids; H atoms are omitted for clarity.

In a seminal study conducted by our group in 2013, a new series of Ru(II) and Os(II) complexes featuring both NHC pincer (C^N^C) and bipyridyl (N^N) ligands was synthesized for exploration of their photophysical properties [17]. These complexes, designated as **[Ru^II^/Os^II^(C^N^C)(N^N)Cl]^+^** (variants **3a**–**3d** and **4a**–**4d**), were prepared by reacting **[Ru^IV^/Os^IV^(N^N)Cl_4_]** with pyridine-bridged bisimidazolium (**^R^CNC^R^**) or bisbenzimidazolium (**^R^C_benz_NC_benz_^R^**) PF_6_ salts. The final complexes were obtained by incorporating C^N^C parts into **[Ru/Os(N^N)Cl]** moieties, followed by Zn-reduction of the high-valent Ru/Os centers to Ru^II^/Os^II^ centers and column chromatography (Scheme 3). Both Ru and Os centers adopt distorted octahedral geometries, with the C^N^C pincer ligand coordinating in a meridional, near-planar fashion. Interestingly, the structural dimensions of the [M-(C^N^C)] fragment were largely unaffected by variations in the C^N^C ligands (**^R^CNC^R^** to **^R^C_benz_NC_benz_^R^**) or the central metal (Ru to Os). For example, the bite angle of the **^R^C_benz_NC_benz_^R^** pincer in the Ru complex **4a** is 156.3(1)°, closely matching that of its Os counterpart **6a**, 155.4(1)°. The Ru−C and Os−C bond lengths were found to be in narrow ranges of 2.033(2)–2.062(4) Å and 2.030(3)–2.040(2) Å, respectively (Figure 3). This consistency in bite angles and bond lengths highlights the rigidity of the C^N^C pincer ligands.

**Scheme 4:**
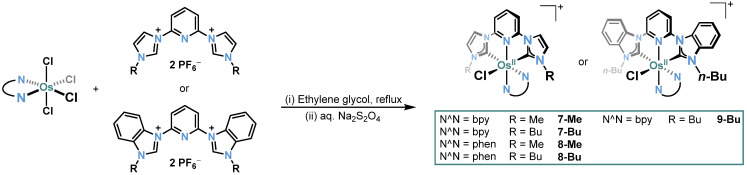
Synthetic routes for **[Os^II^(^Me/Bu^CNC^Me/Bu^)(N^N)Cl]^+^**.

Despite just standing below Ru in the periodic table, Os differs significantly in reactivity, arising from its stronger π-backbonding than Ru to the fixed substrates. Os-NHC complexes are rare, but in 2010, our group first reported Os complexes in the form of **[Os^II^(^Me/Bu^CNC^Me/Bu^)(N^N)Cl]^+^** (N^N = 2,2′-bipyridine, bpy, or 1,10-phenanthroline, phen), prepared by refluxing **[Os^IV^(N^N)Cl_4_]** with CNC-pincer precursors in ethylene glycol and subsequent reduction with aqueous Na_2_S_2_O_4_ in yields of around 70% (Scheme 4) [18]. X-ray crystallography showed that the CNC pincer occupies a meridional coordination site, and the Os center adopts a distorted octahedral geometry. The CNC-pincer bite angle is consistent with its Ru counterpart, again emphasizing the robust coordination mode. Despite Os(II) being known for its π-backdonating capability, the Os–C bond lengths (2.042(4) Å and 2.059(4) Å) suggest a predominantly single-bond character with weak π-backbonding interactions from the Os center to the ligands.

### 2.2. Rh-^R^CNC^R^ Complexes

**Scheme 5:**
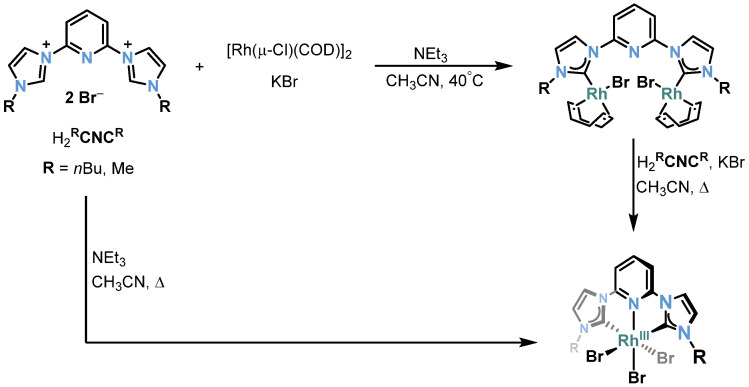
Synthetic routes for **[Rh^III^(^R^CNC^R^)Br_3_]**.

In early 2003, Poyatos et al. explored the coordination chemistry of Rh with CNC-pincer ligands. They reacted dimeric **[Rh^I^(μ-Cl)(COD)]_2_** with equimolar amounts of [H_2_***^n^*^Bu^CNC*^n^*^Bu^**]^2+^ and [H_2_**^Me^CNC^Me^**]^2+^ in CH_3_CN, in the presence of NEt_3_ and KBr, at 40 °C under an inert atmosphere. This reaction yielded a CNC-bridged Rh dimer, [**Rh^I^_2_(*^n^*^Bu/Me^CNC*^n^*^Bu/Me^)(COD)_2_Br_2_]**, with yields ranging from 65% to 80% (Scheme 5). Additionally, the CNC-bridged Rh dimer was taken to react with [H_2_***^n^*^Bu^CNC*^n^*^Bu^**]^2+^ or [H_2_**^Me^CNC^Me^**]^2+^ in refluxing CH_3_CN with KBr to produce a mononuclear Rh(III)-CNC complex (Scheme 5) [19].

**Scheme 6:**
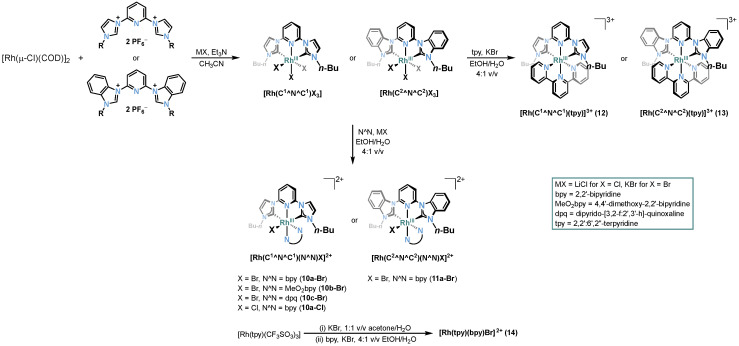
Synthetic routes for **[Rh^III^(C^N^C)(N^N)X]^2+^**; all these complexes were isolated as PF_6_ salts.

In 2020, our group extended the coordination chemistry of Rh-CNC complexes using imidazolium and benzimidazolium-based precursors, [H_2_***^n^*^Bu^CNC*^n^*^Bu^**]^2+^ and [H_2_**^Me^CNC^Me^**]^2+^. Building on Poyatos’ methodology, we took **[Rh^III^(C^N^C)X_3_]** (X = Cl or Br) as molecular templates for incorporating various polypyridyl ligands (Scheme 6). For instance, these templates reacted with 2,2′:6′,2”-terpyridine (tpy) in an EtOH/H_2_O (*v*/*v* = 4:1) mixture, along with KBr, to form heteroleptic Rh-CNC complexes, denoted as **[Rh^III^(C^N^C)tpy]^3+^**, in satisfactory yields (30–70%). Additionally, these templates were allowed to react with electronically diverse bipyridyl ligands under similar conditions to generate **[Rh^III^(C^N^C)(N^N)X]^2+^** (X = Cl or Br; N^N = bpy, 4,4′-dimethoxy-2,2′-bipyridine (MeO_2_bpy), and dipyrido-[3,2-f:2′,3′-h]-quinoxaline (dpq)), with the halide groups determined by the salt used during synthesis [20].

### 2.3. Ir-^R^CNC^R^ Complexes

**Scheme 7:**
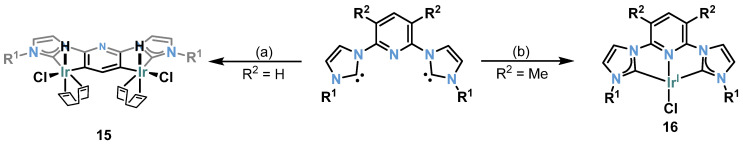
Synthesis of complexes **15** and **16**. Reagents: (a) **[Ir^I^(μ-Cl)(COD)]_2_**, THF, −78 to −30 °C; (b) **[Ir^I^(μ-Cl)(COE)_2_]_2_**, THF, −78 °C to room temperature. R^1^ = 2,6-diisopropylphenyl.

The chemistry of Ir with CNC-pincer ligands is particularly rich, especially with Ir(I) precursors. Danopoulos and co-workers made significant contributions to this field by exploring the reactivity selectivity of Ir(I) precursors toward bisimidazolium salts [21]. They found that reacting **[Ir^I^(μ-Cl)(COE)_2_]_2_** with a bisimidazolium salt, where both meta-positions were methylated, in THF at –78 °C yielded a mononuclear Ir(I)-CNC complex (**16**) (Scheme 7). In contrast, using a pristine bisimidazolium salt with **[Ir^I^(μ-Cl)(COD)]_2_** resulted in a dinuclear complex (**15**) instead of the expected pincer complex. This formation of a dinuclear analogue is likely due to the high tendency of Ir to undergo cyclometallation via C–H activation of aromatic rings.

**Scheme 8:**
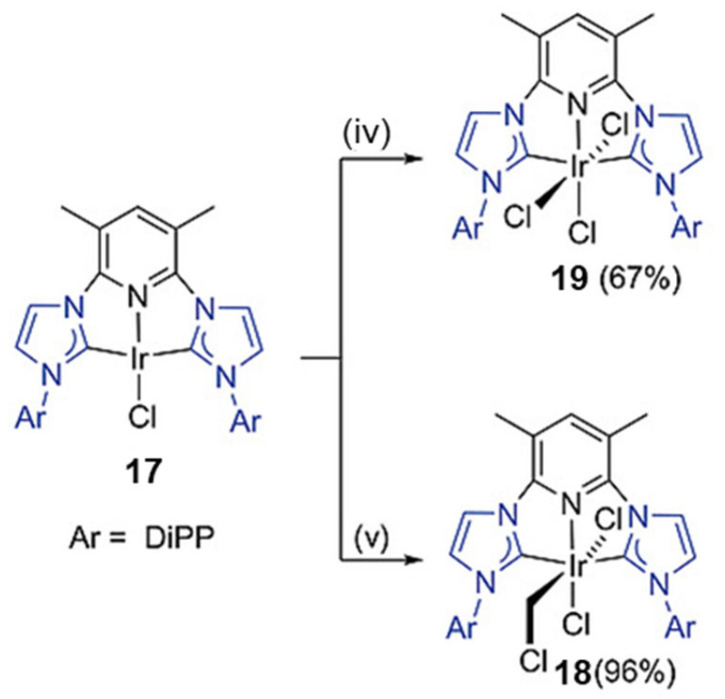
Synthetic routes for Ir^III^ derivatives originating from **17**. Reagents and conditions: (iv) C_6_H_5_ICl_2_ in THF; (v) CH_2_Cl_2_ in THF.

In 2020, the same group began with **[Ir^I^(^DiPP^CNC^DiPP^)Cl]** (**17**) to prepare the Ir(III) counterparts through oxidative addition (Scheme 8). Specifically, **[Ir^I^(^DiPP^CNC^DiPP^)Cl]** was stirred in CH_2_Cl_2_ for one hour, during which the solution changed from green to yellow. The single-crystal structure of the product unveiled the form of **[Ir^III^(^DiPP^CNC^DiPP^)(CH_2_Cl)Cl_2_]** (**18**), highlighting the oxidative addition of CH_2_Cl_2_. **[Ir^III^(^DiPP^CNC^DiPP^)Cl_3_]** (**19**) was obtained if the chlorine surrogate PhICl_2_ was used instead. Despite the angular strain from the pincer’s bite angle, the resulting Ir–C_NHC_ bond lengths were consistent with previously recorded values for Ir^III^–C_NHC_ interactions. Notably, the pyridine component showed a reduced *trans* influence on the chloride ligand compared to the CH_2_Cl group, as reflected by the Ir–Cl bond lengths of 2.3584(11) Å and 2.4783(11) Å, respectively [22].

### 2.4. Ru/Os-^R^CCC^R^ Complexes

**Scheme 9:**
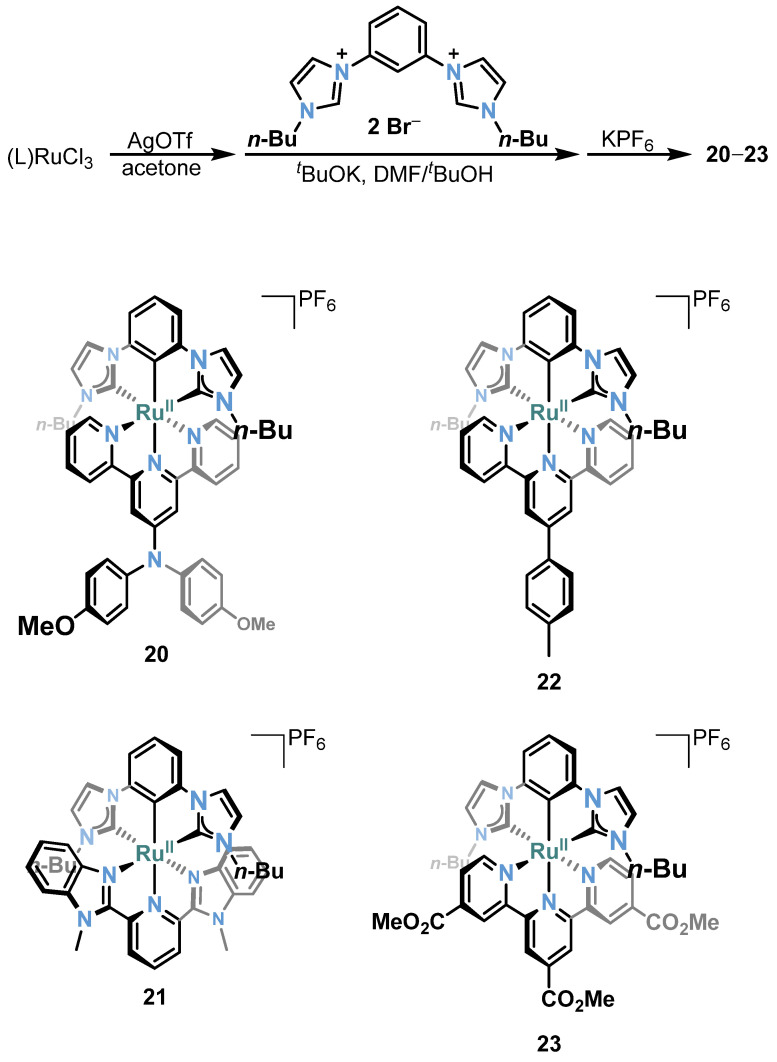
Synthetic routes for complexes **20**–**23**.

**Figure 4:**
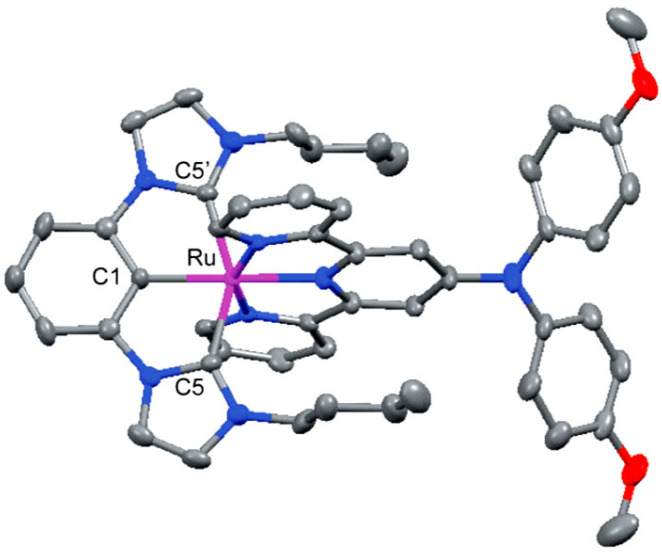
Thermal ellipsoid plot of **20** with 50% probability. H atoms, solvents, and the counteranion are omitted for clarity. Atom color code: carbon, grey; nitrogen, blue; oxygen, red; ruthenium, magenta.

In 2012, Zhong and colleagues introduced the first cyclometalated Ru(II) complexes featuring a CCC-pincer bis-NHC ligand (Scheme 9), thereby creating a versatile platform for the electronic tuning of Ru-based pincers. These air-stable compounds were synthesized using a two-step modular approach. First, (L)RuCl_3_ precursors (L = daatpy, Mebip, ttpy, or Me_3_tcbtpy) underwent chloride abstraction with AgOTf in acetone, forming solvent-coordinated intermediates. Transmetallation with bis-NHC precursor and *t*BuOK produced the target complexes, which were then purified by anion exchange with KPF_6_, yielding compounds **20**–**23** as stable solids of single-crystal quality (Figure 4) in 21–49% yields. This method enabled electronic tuning by selecting ancillary ligands with varying electron-donating (daatpy, Mebip) to electron-withdrawing Me_3_tcbtpy groups [23].

**Scheme 10:**
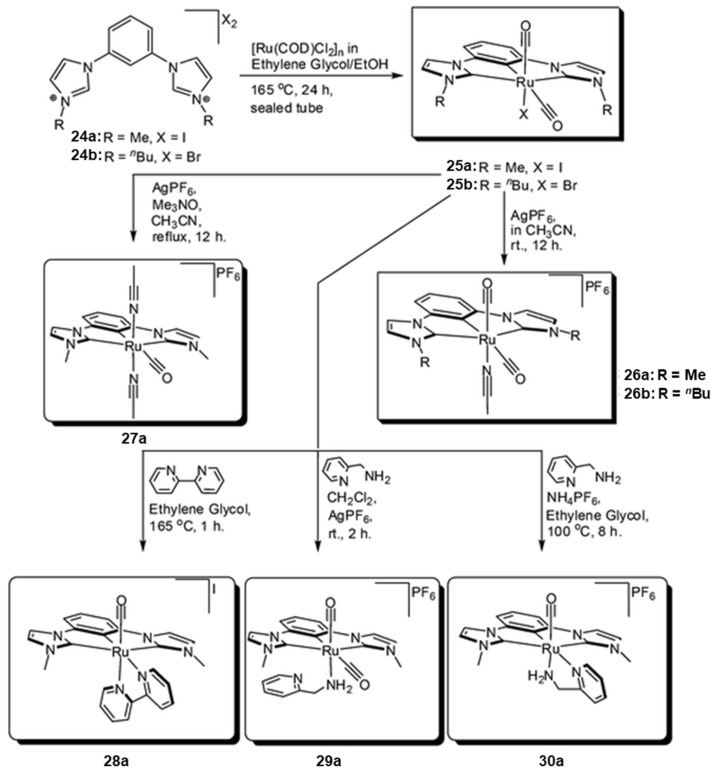
Synthetic routes for CCC-pincer bis-NHC-based Ru(II) complexes (**25**-**30**).

**Figure 5:**
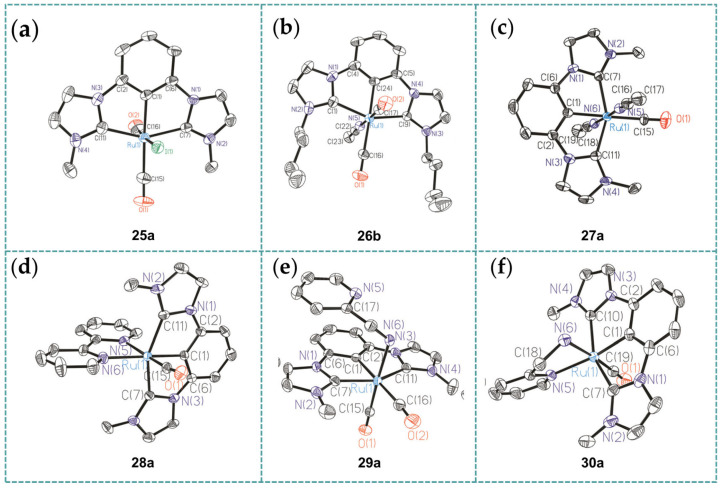
ORTEP plots (30% probability ellipsoids) of (**a**) **25a**, (**b**) **26b**, (**c**) **27a**, (**d**) **28a**, (**e**) **29a**, and (**f**) **30a**. PF_6_ anion and co-solvent (e.g., CH_3_CN, acetone) are omitted for clarity.

In 2013, Naziruddin et al. expanded Ru(II) carbonyl complexes using the same CCC-pincer bis-NHC ligand, creating a versatile platform for catalytic transfer hydrogenation. These air-stable complexes were synthesized through cyclometallation and ligand substitution (Scheme 10). CCC-pincer bis-NHC precursors [H_3_**^R^CCC^R^**]X_2_ (R = Me or *n*Bu; X = I/Br) were reacted with **[Ru(COD)Cl_2_]_n_** in ethylene glycol/EtOH (*v/v* = 1:2) at 165 °C, yielding neutral complexes **[(^R^CCC^R^)Ru^II^(CO)_2_X]** (**25a**: X = I; **25b**: X = Br). CO ligands arose from solvent decarboxylation. Halide abstraction with AgPF_6_ in CH_3_CN produced cationic species **[(^R^CCC^R^)Ru^II^(CO)_n_(CH_3_CN)_3−n_]PF_6_** (**26a**/**26b**, **27a**; n = 1 or 2), and ligand exchange with bidentate amines/imines (e.g., bpy, ampy) led to partially/fully encapsulated amines/imines **[(^R^CCC^R^)Ru^II^(CO)_n_(N^N)]^+^** (**28a**, **29a**, **30a**; n = 1 or 2). Single crystal structures confirmed pseudo-octahedral geometries with meridional pincer coordination (Ru–C_NHC_: 2.07–2.12 Å) (Figure 5). This work highlights the versatility of this synthetic approach for tuning electronic and steric properties in Ru–NHC catalysts [24].

**Scheme 11:**
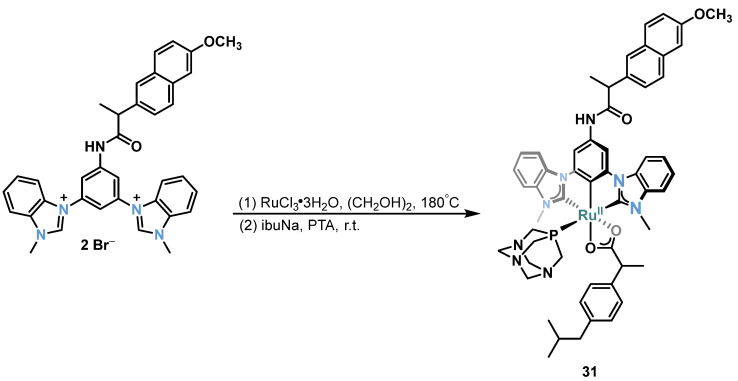
Synthetic route for Ru-CCC complex **31**.

Afterwards, in 2019, Tabrizi and co-workers designed a multifunctional Ru(II) complex that integrates a CCC-pincer bis-NHC ligand with nonsteroidal anti-inflammatory drug (NSAID) pharmacophores through a three-step synthesis. The CCC-pincer precursor was prepared by carbodiimide/hydroxybenzotriazole-mediated amide formation between naproxen and 3,5-dibromoaniline followed by further coupling with 1-methylbenzimidazole (yield: 76%). The target complex, **[Ru(CCC-Nap)(Ibu)(PTA)]** (**31**), was obtained as air-stable orange crystals (yield: 68.5%) by refluxing RuCl_3_•3H_2_O with the CCC-pincer precursor in ethylene glycol and subsequent chelation by ibuprofen salt (Ibu^−^) and 1,3,5-triaza-7-phosphaadamantane (PTA) (Scheme 11). Crystallographic analysis of **31** revealed a pseudo-octahedral geometry with Ru-C_NHC_ bond lengths (2.006–2.034 Å) and a bite angle (151.4°), resembling classical C^N^C pincers. The Ru-O_Ibu_ (2.258–2.265 Å) and Ru-P_PTA_ (2.599 Å) distances were consistent with literature values, highlighting the scaffold’s adaptability for NSAID ligands while preserving structural integrity—key for COX-2 inhibition and cytotoxicity [25].

**Scheme 12:**
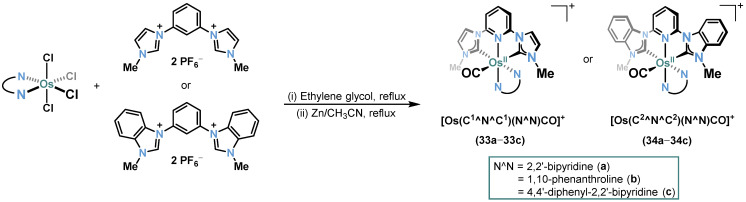
Synthetic routes for **[Os^II^(C^C^C)(N^N)(CO)]^+^** (**32**-**33**) and its synthetic route.

**Figure 6:**
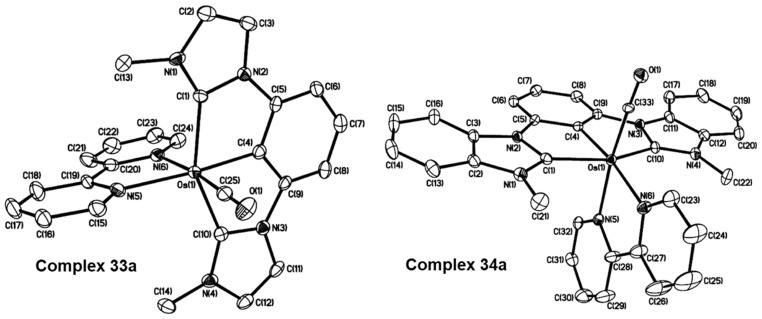
Perspective views of **33a** and **34a**. H atoms are omitted for clarity. Thermal ellipsoids are at the 30% probability level.

Early in 2012, our group developed two CCC-pincer bis-NHC ligands: 1,3-bis(1-methylimidazolin-2-ylidene)phenyl anion (C^1^^C^C^1^) and 1,3-bis(3-methylbenzimidazolin-2-ylidene)phenyl anion (C^2^^C^C^2^). Upon refluxing in ethylene glycol, these CCC-pincer precursors were deprotonated and chelated **[Os^IV^(N^N)Cl_4_]** (N^N = bpy, phen, Ph_2_bpy) (Scheme 12). Then, the isolated intermediates were allowed to undergo reductive carbonylation in CH_3_CN with Zn and transformed into air-stable complexes **[Os^II^(C^C^C)(N^N)(CO)]^+^** (**33**–**34**). As reflected by the single crystal structure (Figure 6), similar to Ru(II) analogues, the Os(II) center adopted a distorted octahedral geometry with meridional C^C^C coordination (bite angles: 152–153°) and Os–C_NHC_ bond lengths (2.079–2.103 Å). Spectroscopic analyses (^13^C NMR: Os–C_NHC_ signals at 172–185 ppm; IR: ν_CO_ = 1906–1927 cm^−1^) showed the stronger σ-donating power of C^1^^C^C^1^ than C^2^^C^C^2^. This work presents the first example of Os(II) complexes bearing a CCC-pincer bis-NHC ligand and lays the synthetic foundation for upcoming Os(CCC-pincer-NHC) complexes [26].

**Scheme 13:**
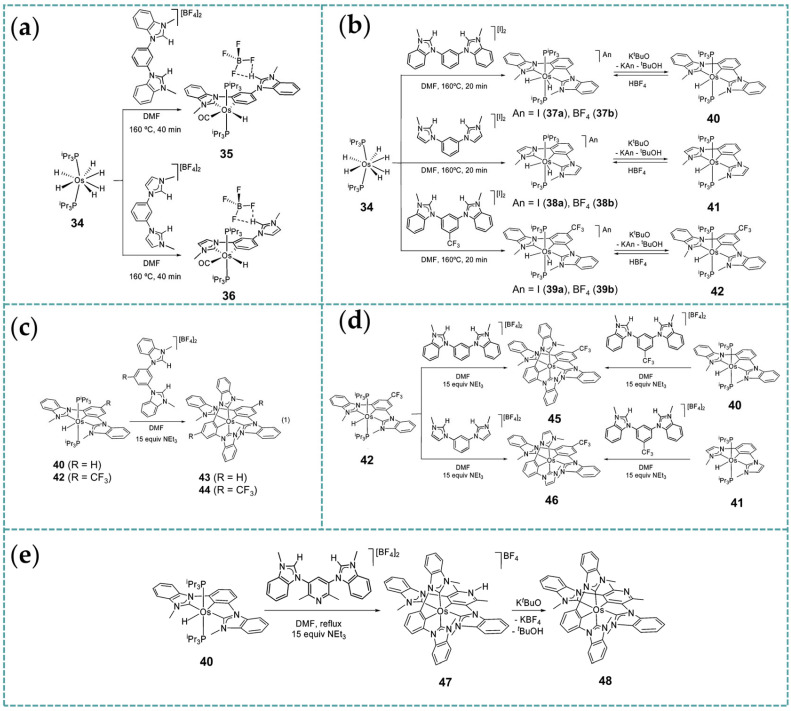
Synthetic routes for CCC-pincer bis-NHC-based Os(II) complexes (**35**-**48**). Reaction schemes for Os(II) complexes (**a**) **35**-**36**, (**b**) **40**-**42**, (**c**) **43**-**44**, (**d**) **45**-**46** and (**e**) **47**-**48**.

**Figure 7:**
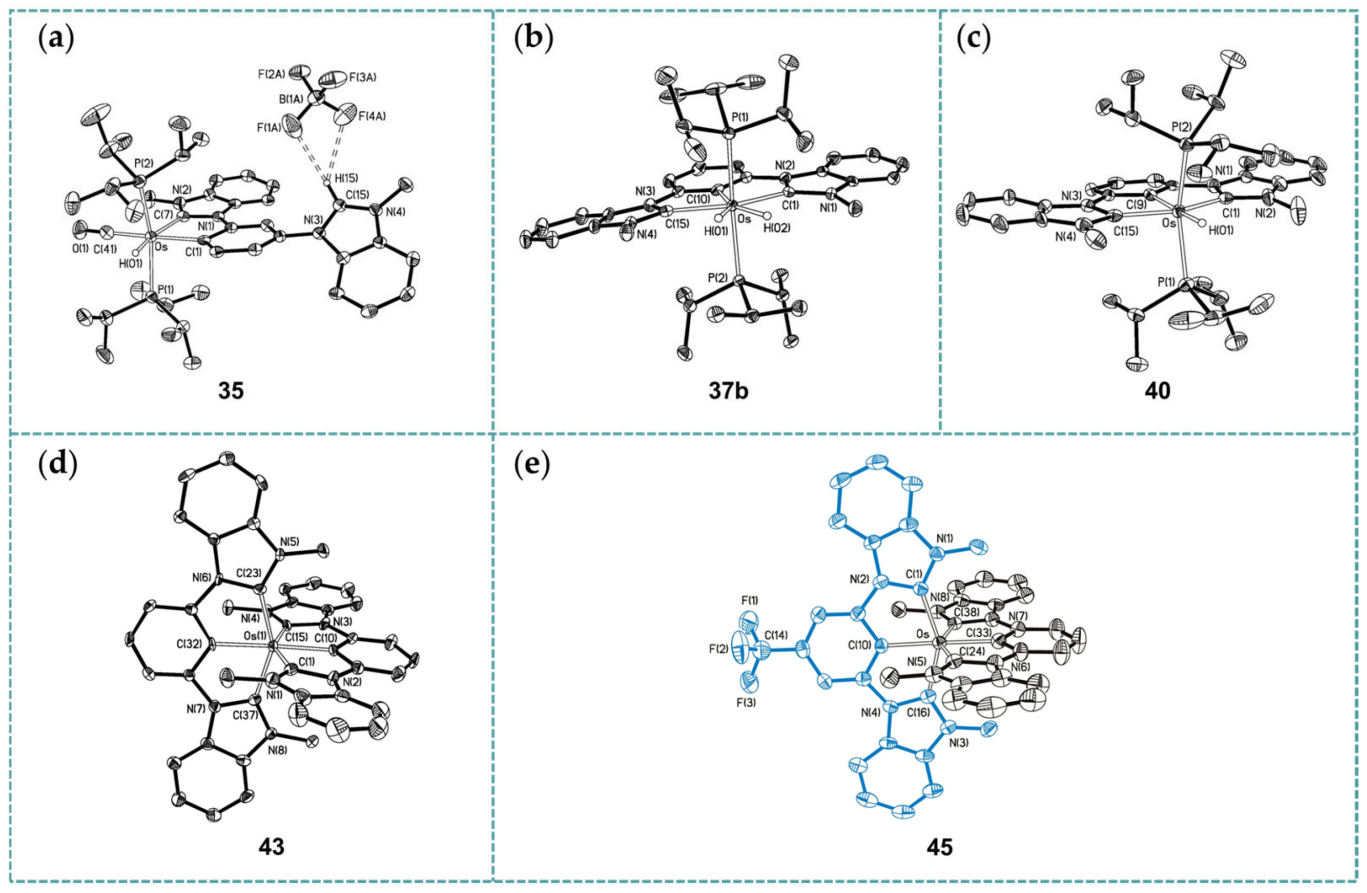
(**a**) ORTEP diagrams of (**a**) complex **35**, (**b**) **37b**, (**c**) **40**, (**d**) **43**, and (**e**) **45** (50% probability ellipsoids); H atoms (except the hydride and hydrogen-bonded H) are omitted for clarity.

Two years later, Esteruelas et al. reported a novel series of Os(II) complexes with CCC-pincer bis-NHC ligands (C_NHC_C_aryl_C_NHC_) for blue–green emissive OLED applications. The complexes, including homoleptic **[Os^II^(C_NHC_C_aryl_C_NHC_)_2_]** (**43**, **44**), heteroleptic **[Os^II^(C_NHC_C_aryl_C_NHC_)(C_NHC_C_aryl‘_C_NHC_)]** (**45**, **46**), and pyridinium-bridged variants (**47**, **48**), were synthesized via a modular strategy starting from the hexahydride precursor **[Os^VI^H_6_(P^i^Pr_3_)_2_]** (**34**) (Scheme 13). Reactions with bis(benzimidazolium/imidazolium) salts and anion-controlled conditions (BF_4_^−^ vs. I^−^) yielded either Os(II) hydride-carbonyl intermediates (**35**, **36**) or Os(IV) dihydrides (**37a**–**39a**). Deprotonation of Os(IV) dihydrides with KO*t*Bu produced Os(II) monohydrides (**40**–**42**), which underwent ligand exchange to form the final bis(tridentate) complexes. All complexes exhibited distorted octahedral geometries, with rigid five-membered metallacycles, consistent Os–C bond lengths (2.030–2.087 Å), and C_NHC_-Os-C_NHC_ bite angles (~150°) (Figure 7). Notably, heteroleptic variants (e.g., **45**) featured aryl and trifluoromethyl-substituted aryl bridges, while pyridinium-bridged **47** (later deprotonated to **48**) introduced heterocyclic diversity. This approach enabled tuning of photophysical properties, achieving high quantum yields (up to Φ = 0.62 for **44**) and narrow emission profiles, crucial for OLED performance [27].

### 2.5. Rh-^R^CCC^R^ Complexes

**Scheme 14:**
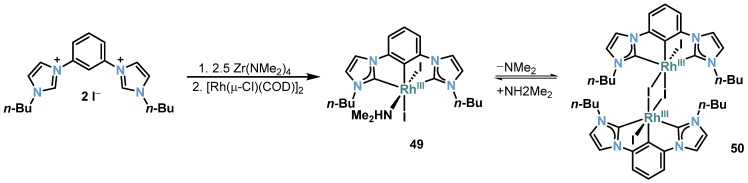
Synthetic route for CCC-pincer bis-NHC-based Rh(III) complexes **49** and **50**.

**Figure 8:**
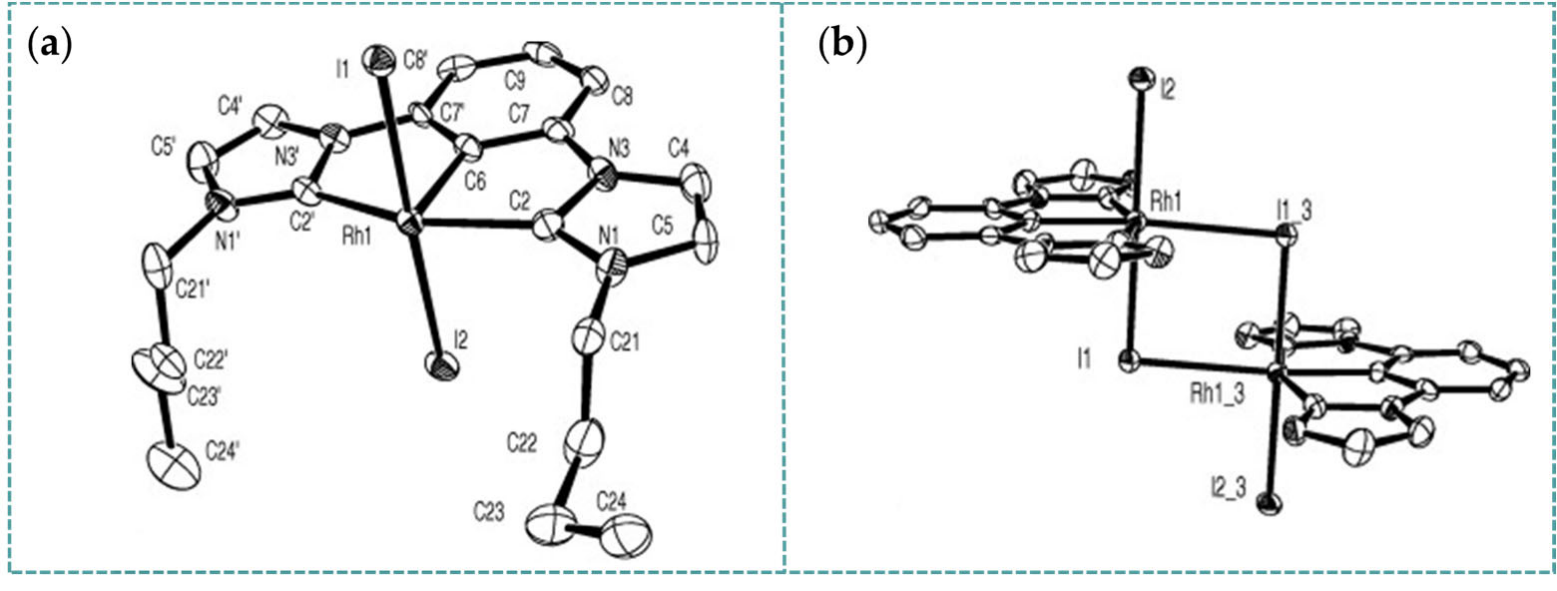
(**a**) ORTEP plot (50% probability, H atoms omitted for clarity) of the asymmetric unit of **14**; (**b**) ORTEP plot (50% probability, H and C atoms of the butyl groups omitted for clarity) of the edge-on view of the dimer (**13**) structure (Rh-Rh = 4.01 Å).

As early as 2005, Rubio et al. developed a transmetallation strategy to synthesize CCC-NHC Rh pincer complexes. The Zr precursor, formed by reacting 1,3-bis(*N*-butylimidazolium)benzene diiodide with Zr(NMe_2_)_4_, was treated with **[Rh^I^(μ-Cl)(COD)]_2_** in CH_2_Cl_2_ under an inert atmosphere (Rh:ligand = 2:1), yielding the dinuclear Rh(III) complex **50** (yield: 66%). Under the action of NHMe_2_, **50** reversibly converted back to mononuclear **49** (Scheme 14). X-ray structural analysis (Figure 8) revealed that **50** adopts a μ-iodide-bridged dimeric structure (space group: *P*2_1_/*c*), with each Rh center coordinating a tridentate CCC-NHC ligand and three iodide ligands, forming a distorted octahedral geometry (Rh–C_NHC_: 2.06 Å; Rh–C_aryl_: 1.94 Å; Rh–I_bridge_: 2.84–2.87 Å). This work demonstrates the effectiveness of transmetallation from early to late transition metals in constructing stable NHC-based pincer architectures [28].

**Scheme 15:**
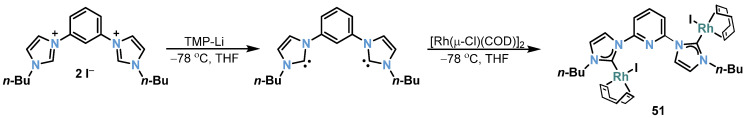
Synthetic route for the di-Rh complex **51**.

**Figure 9:**
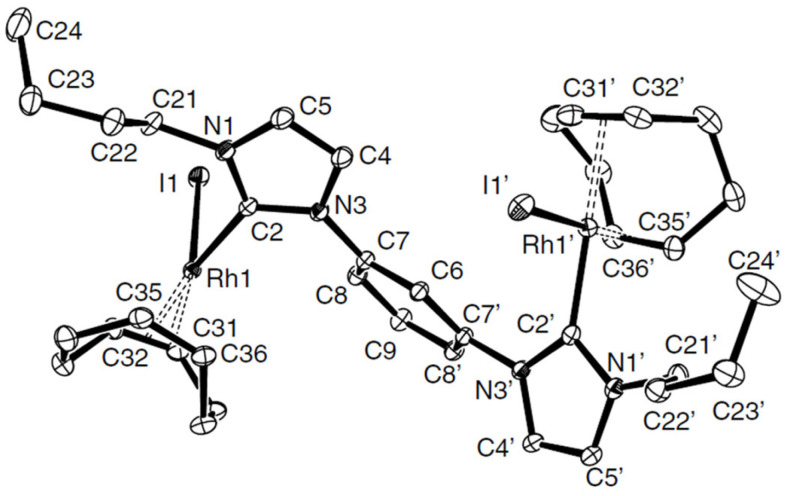
ORTEP diagram of **51** (50% probability, H atoms are omitted for clarity).

In the same year, Andavan et al. developed a one-step direct coordination method where lithium tetramethylpiperidine (TMP-Li) was used to deprotonate the imidazolium salt at −78 °C to generate a free bis-NHC intermediate, which reacted directly with **[Rh^I^(μ-Cl)(COD)]_2_** to form a dinuclear Rh(I) bis-NHC complex (**51**) (Scheme 15). This complex features a benzene-bridged structure, with each Rh center coordinating one NHC, one COD ligand, and one iodide under low-temperature conditions. In contrast, Rubio’s Rh(III) pincer complex, as revealed by X-ray crystallography, exhibited a μ-iodide-bridged dinuclear structure (Rh–Rh = 4.01 Å) and a distorted octahedral geometry (Rh–C_NHC_ = 2.06 Å, Rh–C_aryl_ = 1.94 Å), showing no dynamic isomerism. Andavan’s complex, however, adopted a square-planar geometry (Rh–C_NHC_ = 2.015–2.020 Å) with diastereomerism in solution (Figure 9), as evidenced by the splitting of NMR signals due to hindered rotation of the benzene bridge. Crystallization produced an enantiomeric pair (*R*, *R*/*S*, *S*), related by an inversion center. The sterically bulky benzene bridge influenced both isomerization dynamics and catalytic behavior, modulated by additives such as LiCl [29].

**Scheme 16:**
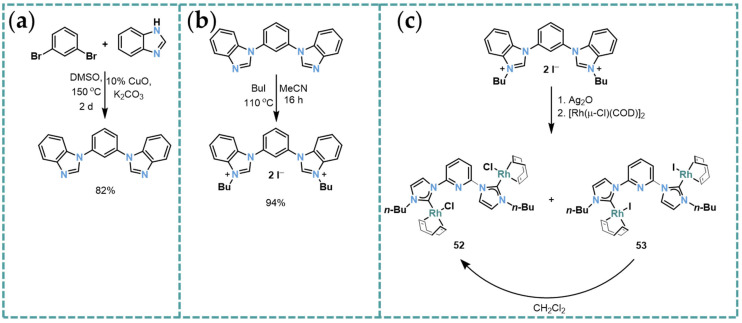
(**a**) Synthesis of phenylene-bridged bisbenzimidazole by Cu-catalyzed *N*-arylation of benzimidazole with 1,3-dibromobenzene; (**b**) synthesis of phenylene-bridged bisimidazolium by butylation of phenylene-bridged bisbenzimidazole; (**c**) formation of Rh complexes **52** and **53**.

**Figure 10:**
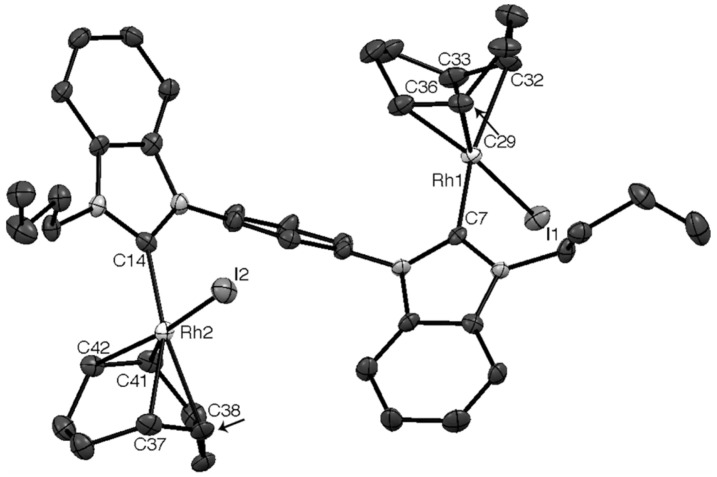
ORTEP plot of the crystallographically determined structure of **53** (thermal probability ellipsoids drawn at the 50% confidence level). H atoms are omitted for clarity.

In 2012, Hollis et al. utilized 1,3-phenylene-bridged bis(*N*-butylbenzimidazolium) diiodide as a CCC-pincer bis-NHC ligand precursor, which upon treatment with Ag_2_O in CH_3_CN, generated a dinuclear Ag(I)-NHC complex, confirmed by ESI-TOF MS (*m/z* 955.5). Transmetallation with **[Rh^I^(μ-Cl)(COD)]_2_** or **[Rh^I^(COD)_2_OTf]**/NBu_4_I produced chloride- (**52**) and iodide-ligated (**53**) dinuclear Rh(I) complexes, with **53** selectively obtained (51% yield) under chloride-free conditions (Scheme 16). The single crystal structure of **53** revealed a square-planar geometry with Rh–C (2.001–2.010 Å) and Rh–I (2.6776–2.6811 Å) bond lengths (Figure 10). The benzimidazole rings twisted 60.7° relative to the phenylene bridge, inducing spontaneous chiral resolution (space group *P*2_1_). Solution-phase studies identified diastereomerism (*rac*/*meso*) due to restricted rotation of the phenylene bridge, as shown by splitting ^1^H NMR signals (*rac*-H_a_ δ 7.95, *meso*-H_a_ δ 8.10). The iodide complex **53** exhibited slower isomerization dynamics than **52**, highlighting steric modulation by the bulky iodide ligand [30].

**Scheme 17:**
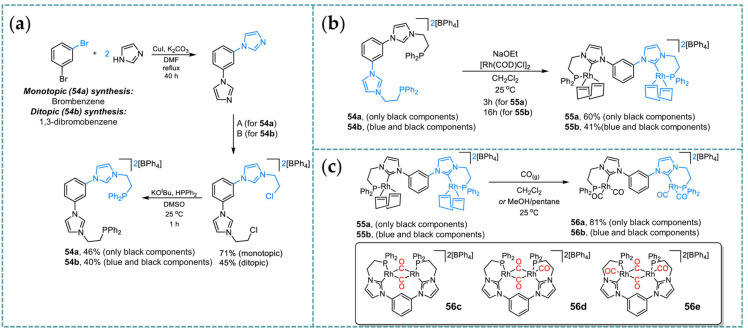
(**a**) Synthetic routes for proligands **54a** and **54b**, (**b**) **[Rh^I^(COD)(NHC)]** complexes **55a** and **55b**, and (**c**) the bis-CO-substituted Rh(I) complexes **56a** and **56b**.

**Figure 11:**
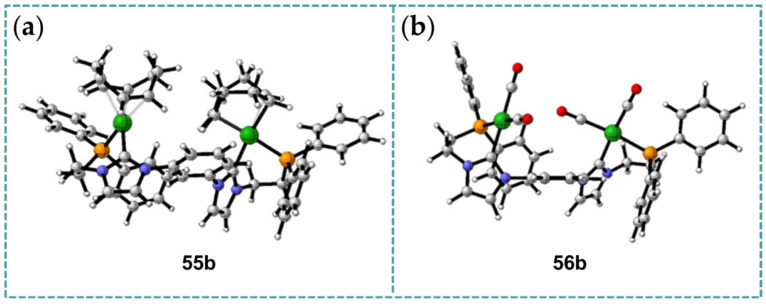
Lowest energy conformations of complexes (**a**) **55b** and (**b**) **56b**, calculated at CPCM (THF) M06-D3/def2TZVP//ωB97X-D/6-31G(d)+SDD.

Bimetallic complexes often outperform monometallic counterparts in organic transformations due to their superior activity and selectivity. Based on the previous work with NHC/pyrazole-ligated Rh complexes by Messerle et al., Pernik and co-workers replace pyrazole with electron-deficient phosphine ligands to prepare mono- and bimetallic Rh(I) complexes for hydrosilylation catalysis. The ligand design features mono- (**54a**) and ditopic (**54b**) NHC/phosphine scaffolds synthesized via Ullmann coupling (Scheme 17a). The monodentate ligand was made from bromobenzene in 1,2-DCE at 120 °C for 16 h, while the ditopic ligand was prepared from 1,3-dibromobenzene in DMF/1,2-DCE (*v/v* = 3:1) for a longer heating duration (48 h) to minimize side reactions. After alkylation and anion exchange with Na[BPh_4_], both ligands exhibiting comparable environments were obtained (^31^P singlets at −21.9 ppm for **54a** and −22.1 ppm for **54b**). Coordination with **[Rh^I^(μ-Cl)(COD)]_2_** in CH_2_Cl_2_, aided by NaOEt, produced monometallic **55a** (3 h) and bimetallic **55b** (16 h) (Scheme 17b), verified by ^31^P NMR doublets (24.3 ppm, ^1^*J*_Rh-P_ = 154 Hz for **55a**; 23.4 ppm, ^1^*J*_Rh-P_ = 153 Hz for **55b**) and dynamic behavior in variable-temperature NMR. Replacing COD with CO in a CO atmosphere produced monometallic **56a** and bimetallic **56b** (Scheme 17c), with **56b** rapidly forming a bridging CO intermediate (IR: 1995 cm^−1^), which is energetically favored (ΔG = −15.5 kcal mol^−1^ vs. terminal CO), though unstable at low CO concentrations (decomposing to [(M−2CO−H)]^+^, M = cationic fragment of **56b**). ESI-MS/MS, HRMS, and DFT-optimized geometries (Rh–Rh distance ~5 Å) (Figure 11) revealed that bimetallic enhancement results from proximity-induced ligand destabilization, promoting precatalyst activation via COD dissociation and silane-mediated reduction to cyclooctane. This strategy which merges controlled intermetal spacing with electron-poor phosphines sets a framework for rational bimetallic catalyst design [31].

### 2.6. Ir-^R^CCC^R^ Complexes

**Scheme 18:**
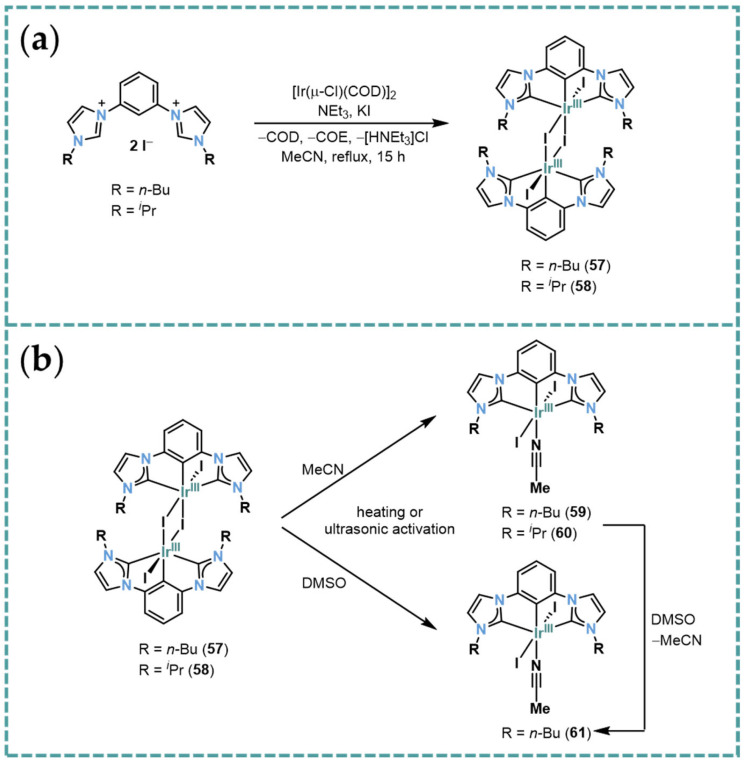
(**a**) One-pot synthesis of Ir(III) CCC-pincer bis-NHC complexes (**57**-**58**). (**b**) Route showing solvolysis of iodine bridges of **57**-**58**.

**Figure 12:**
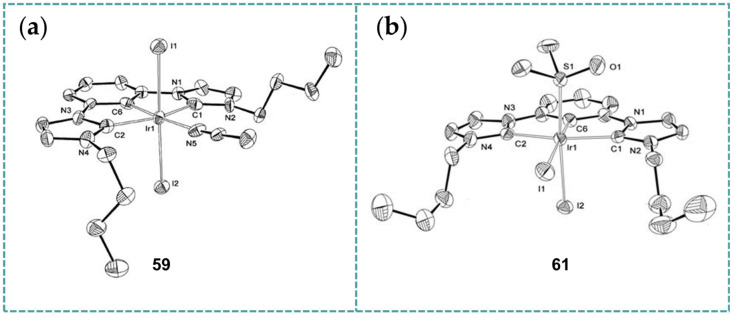
(**a**) ORTEP plot of the molecular structure of **59** in **59**•MeCN. H atoms omitted for clarity. Ellipsoids are represented at a 50% probability level; (**b**) ORTEP plot of the molecular structure of one of the two crystallographically independent molecules of **61** in **61**•1/2CH_2_Cl_2_. H atoms omitted for clarity.

**Scheme 19:**
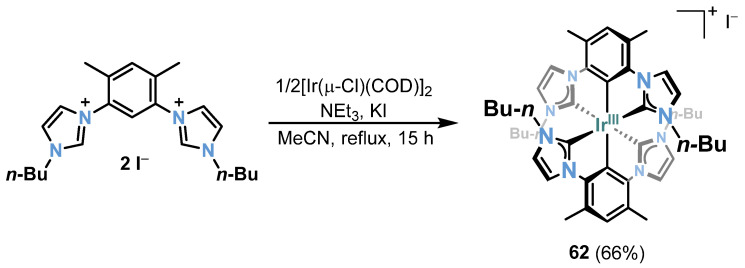
Synthetic route for complex **62**.

**Figure 13:**
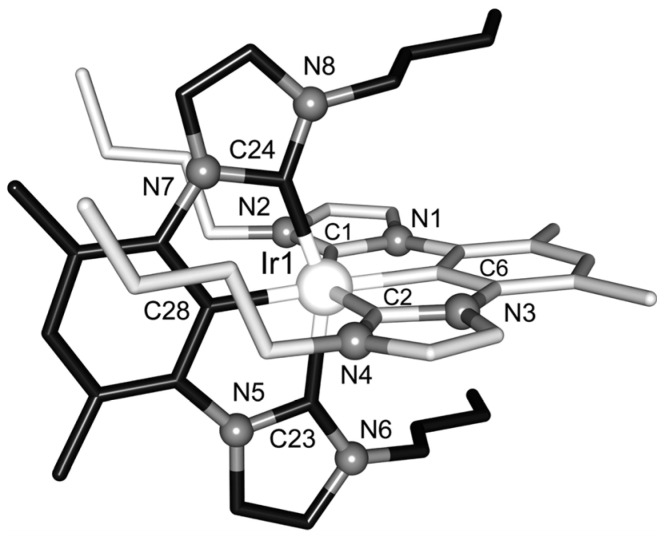
The molecular structure of the cation in **62**•2CH_2_Cl_2_.

The synthesis of iridium-based pincer complexes has long been limited to inefficient and cumbersome indirect methods such as transmetallation via zirconium intermediates. Until 2008, Braunstein et al. reported a one-pot synthesis of Ir(III) CCC-pincer bis-NHC complexes by reacting bis(imidazolium) precursors with the Ir(I) precursor **[Ir^I^(μ-Cl)(COD)]_2_** in the presence of NEt_3_ and KI (Scheme 18a). This direct approach led to the formation of novel dinuclear pincer complexes **[Ir^I^_2_(μ-I)_2_(COD)_2_(C_NHC_CC_NHC_)]** (**57**, **58**) without transmetallation. Treatment with solvents like MeCN or DMSO cleaved the iodide bridges, yielding mononuclear solvent adducts (**59**–**61**) (Scheme 18b). The coordination mode of the solvent—CH_3_CN *trans* to the aryl group vs. DMSO *trans* to iodides—significantly influenced the reactivity (Figure 12). This streamlined method simplifies the synthesis and modulates coordination environments for catalytic applications. Later, the same group successfully prepared the first Ir(III) complex bearing bis-CCC-pincers, **62**, by reacting the 4,6-dimethyl-substituted bis(imidazolium) salt with **[Ir^I^(μ-Cl)(COD)]_2_** in CH_3_CN, using NEt_3_ as the base and KI for halide exchange (Scheme 19). The Ir(III) center was coordinated by NHC on both sides, with the aryl ring metallating at the C2 position to form the bis-pincer structure (Figure 13). This strategy, assisted by the steric effect of methyl substituents and excess base, provided an efficient route for high-symmetry bis-pincer iridium complexes [32,33].

**Scheme 20:**
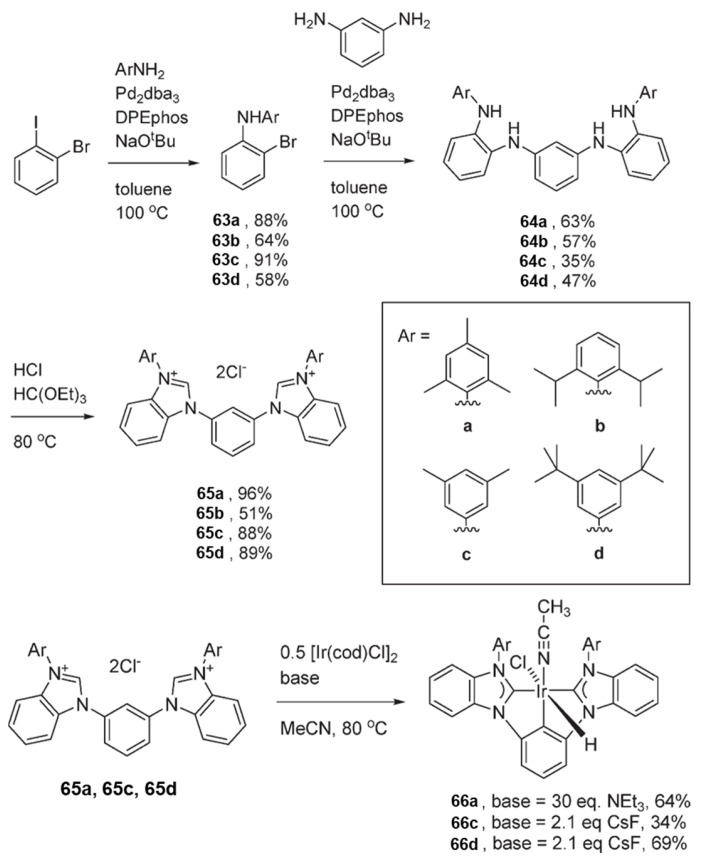
Synthetic routes for Ir(III)-hydride complexes **66a**, **66c**, and **66d**.

**Figure 14:**
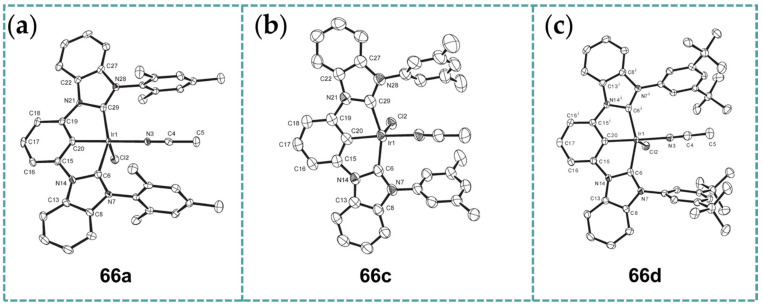
ORTEP diagrams of (**a**) **66a**, (**b**) **66c**, and (**c**) **66d**, showing 50% probability ellipsoids with H atoms omitted for clarity.

In 2010, Chianese et al. reported Ir(III) complexes bearing CCC-pincer bis-NHC ligands decorated with bulky aromatic substituents. They synthesized a series of four meta-phenylene-bridged bis-benzimidazolium chlorides (**65a**–**d**) with varying aryl groups (e.g., mesityl, 3,5-xylyl, and 3,5-di-*tert*-butylphenyl) via Buchwald–Hartwig amination, palladium-catalyzed coupling, and cyclization (Scheme 20). Neutral Ir(III) complexes (**66a**, **66c**, **66d**), of the formula **[Ir^III^(CCC)(H)(Cl)(NCCH_3_)]**, were obtained by reacting the ligand precursors with **[Ir^I^(μ-Cl)(COD)]_2_** in CH_3_CN, using NEt_3_ or CsF as a base. Metallation was unsuccessful for the 2,6-diisopropylphenyl-substituted derivative (**65b**), likely due to significant steric hindrance. X-ray crystallography revealed that all Ir(III) complexes adopt an octahedral geometry, with CH_3_CN standing *trans* to the central aryl carbon (C_aryl_), and the hydride and chloride ligands staying *trans* to each other (Figure 14). The Ir–C_aryl_ bond lengths (1.944–1.971 Å) are shorter than those in traditional PCP-pincer complexes, due to the rigid CCC-pincer backbone. The design of these rigid CCC-pincer bis-NHC ligands enhances catalytic performance by combining the strong σ-donor of NHCs with the geometric constraints of the pincer structure. On the other hand, bulky aryl substituents, like mesityl groups, protect iridium intermediates from aggregation and deactivation. This work lays the foundation for developing robust catalysts for C–H functionalization, such as alkane dehydrogenation and arene borylation [34].

**Scheme 21:**
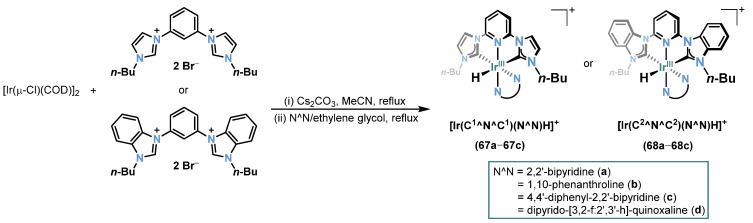
Synthetic route for **[Ir^III^(C^C^C)(N^N)(H)]^+^**.

**Figure 15:**
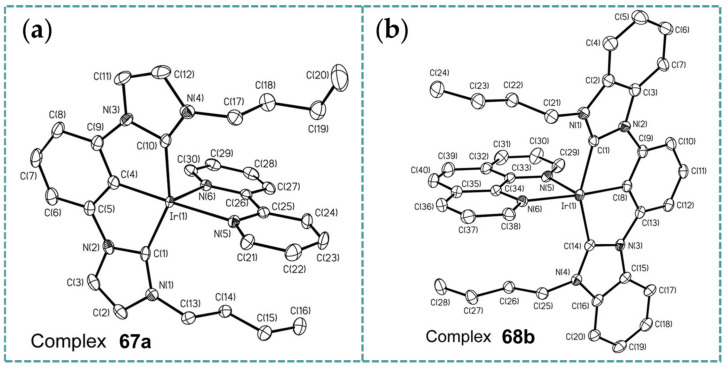
Perspective views of complexes (**a**) **67a** and (**b**) **68b**; thermal ellipsoids are at the 50% and 30% probability levels, respectively. H atoms are omitted for clarity.

In 2015, our group reported a series of Ir(III) hydrido complexes supported by NHC-based C^C^C-pincer ligands and N^N in the form of **[Ir^III^(C^C^C)(N^N)(H)]^+^**, focusing on their photophysical properties and ligand-mediated luminescent regulation. Neutral Ir(III) hydrido intermediates, **[Ir^III^(C^C^C)(CH_3_CN)(Br)(H)]**, were synthesized by reacting benzene-bridged bisimidazolium or bisbenzimidazolium salts (e.g., 1,3-bis(1-butylimidazolin-2-ylidene)phenyl anion (C^1^^C^C^1^) and 1,3-bis(3-butylbenzimidazolin-2-ylidene)phenyl anion (C^2^^C^C^2^)) with **[Ir^I^(μ-Cl)(COD)]_2_** (Scheme 21). Refluxing these intermediates with N^N (e.g., bpy, phen, Me_2_bpy, and dipyrido[3,2-f:2′,3′-h]quinoxaline (dpq)) in ethylene glycol yielded the target complexes **[Ir^III^(C^C^C)(N^N)(H)]^+^** (**67**–**68**), isolated as ClO_4_^−^ salts through anion exchange. X-ray crystallography revealed that these Ir(III) complexes have a distorted octahedral geometry, with the C^C^C-pincer ligands adopting a planar meridional coordination mode (Figure 15). The Ir–C_aryl_ bond lengths (1.959–1.986 Å) were shorter than the Ir–C_NHC_ bond lengths (2.043–2.056 Å), due to the rigid C^C^C-pincer framework [35].

**Figure 16:**
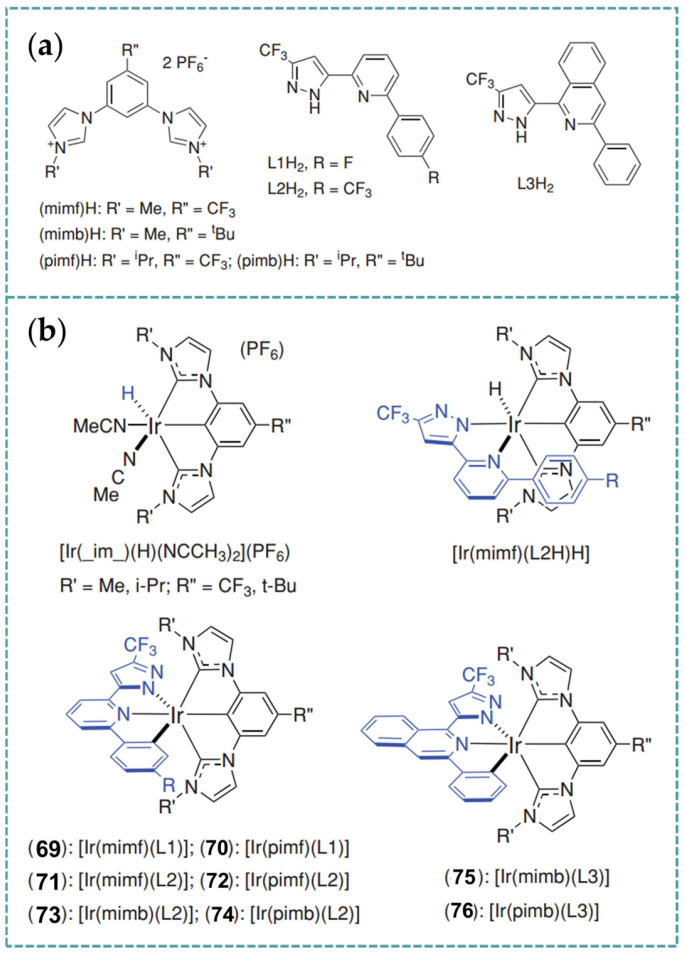
(**a**) Structures of cyclometallating tridentate chelates. The first and last letters of notation around “im” indicate methyl (m), *i*-propyl (p), trifluoromethyl (f), and *t*-butyl (b) on the respective *N*-imidazolyl and central benzene unit; (**b**) structures of Ir(III) complexes bearing bis-tridentate pincers (**69**-**76**).

NHC ligands, known for their strong σ-donating ability, enhance metal–ligand bond strength and reduce non-radiative decay pathways. However, previous studies on NHC-based Ir(III) complexes have primarily focused on ionic species, which limits their use in device fabrication. To address this, neutral, vacuum-depositable Ir(III) phosphors were developed by designing bis-tridentate pincer ligands that increase structural rigidity and optimize excited-state properties, such as the MLCT transitions. Chou and co-workers introduced a novel class of bis-tridentate Ir(III) complexes with NHC-based C^C^C pincer ligands and N^N^C (Figure 16). The synthesis was achieved in a two-step fashion: first, Ir(III) hydride intermediates **[Ir^III^(C^C^C)(H)(NCCH_3_)_2_]^+^** were prepared and allowed to react with N^N ligands (e.g., 2-pyrazolyl-6-phenylpyridine derivatives) to form bis-tridentate complexes like **[Ir(mimf)(L2)]** (**71**). Benzene-bridged bisimidazolium salts (e.g., **mimf**, **mimb**, **pimf**, **pimb**) were used, with methyl or isopropyl groups fine-tuning steric bulkiness and CF_3_/*tert*-butyl groups regulating electron density, suppressing cyclometallation at non-target sites. X-ray crystallography revealed that these Ir(III) complexes take up an octahedral geometry, with shorter Ir–C_aryl_ bonds (1.959–1.986 Å) than Ir–C_NHC_ bonds (2.043–2.056 Å), attributed to the rigid pincer framework [36].

## 3. Photophysical Property of d^6^ Transition Metal-NHC Complexes

### 3.1. Photophysical Property of Ru/Os-CNC Pincer Complexes

**Figure 17:**
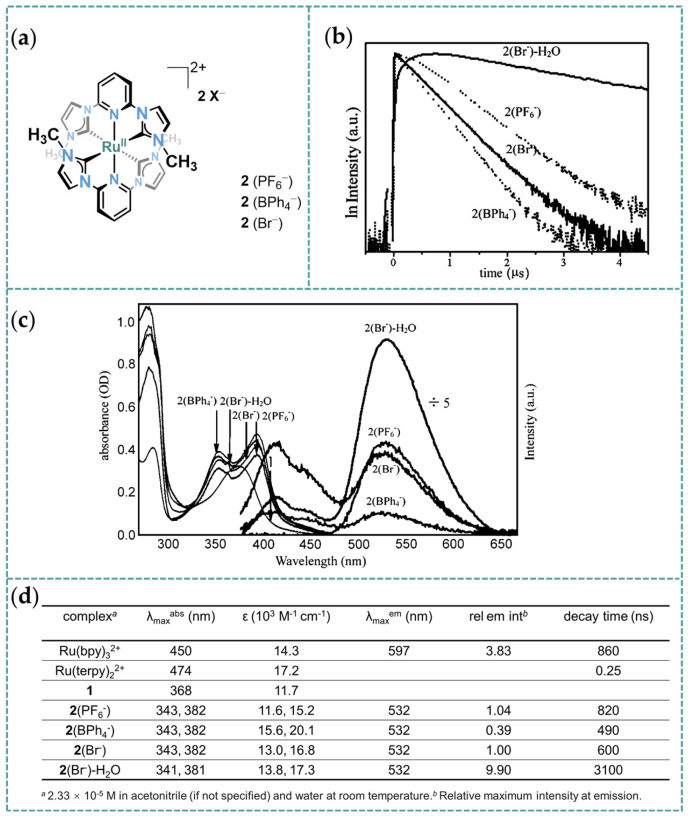
(**a**) **[Ru^II^(^Me^CNC^Me^)_2_]^2+^** complex; (**b**) emission kinetic curves excited at 355 nm and monitored at 532 nm, of Ru(II) complexes in CH_3_CN (if not specified) and H_2_O under Ar; (**c**) absorption and emission spectra, with excitation at 354 nm, of **[Ru^II^(^Me^CNC^Me^)_2_]^2+^** complexes in CH_3_CN (if not specified) and H_2_O under Ar; (**d**) spectroscopic properties of the selected Ru(II) complexes (**1** = **[**Ru(C^N)_3_**]^2+^**, C^N represents pyridylimidazol-2-ylidene).

**[Ru^II^(^Me^CNC^Me^)_2_]^2+^** (Figure 17a) stands out as a promising candidate in the study of photophysical properties, exhibiting significant divergence from the absorption characteristics of classical **[Ru^II^(tpy)_2_]^2+^**. The complex has two prominent absorption bands at 343 nm and 382 nm, which are notably blue-shifted compared to the moderately intense bands of **[Ru^II^(bpy)_3_]^2+^** and **[Ru^II^(tpy)_2_]^2+^** (cf. λ_abs_ centered at 450 and 474 nm, respectively, Figure 17c). Preliminary ab initio computational analysis of **[Ru(^Me^CNC^Me^)_2_]^2+^** suggests that these blue-shifted peaks result from the d*π*(Ru^II^) → π*(^Me^CNC^Me^) ^1^MLCT transitions. In stark contrast to **[Ru^II^(tpy)_2_]^2+^**, **[Ru^II^(^Me^CNC^Me^)_2_]^2+^** exhibited robust orange photoluminescence (λ_em_ centered at 532 nm, Figure 17c). This emission was systematically investigated by varying the excitation wavelengths, with the emission profile closely mirroring the absorption spectrum. This confirmed that the observed luminescence at 532 nm is an intrinsic property of **[Ru^II^(^Me^CNC^Me^)_2_]^2+^** and not due to impurities. The photoluminescence kinetics of **[Ru^II^(^Me^CNC^Me^)_2_]^2+^**, shown in Figure 17b, revealed an extended lifetime of 820 ns in CH_3_CN at ambient temperature, which is three orders of magnitude longer than the brief 0.25 ns lifetime of **[Ru^II^(tpy)_2_]^2+^**. Given the importance of chromophore longevity in artificial photosynthesis, **[Ru^II^(^Me^CNC^Me^)_2_]^2+^** was synthesized with bromide as the counterion to evaluate photophysical stability in aqueous environments. Remarkably, the excited-state lifetime of **[Ru^II^(^Me^CNC^Me^)_2_]^2+^** was 3100 ns in H_2_O, a duration over 12,000 times longer than that of **[Ru^II^(tpy)_2_]^2+^** (Figure 17d). This positions **[Ru^II^(^Me^CNC^Me^)_2_]^2+^** among the top luminescent lifetimes of **[Ru^II^(tpy)_2_]^2+^** derivatives in aqueous media [37].

**Figure 18:**
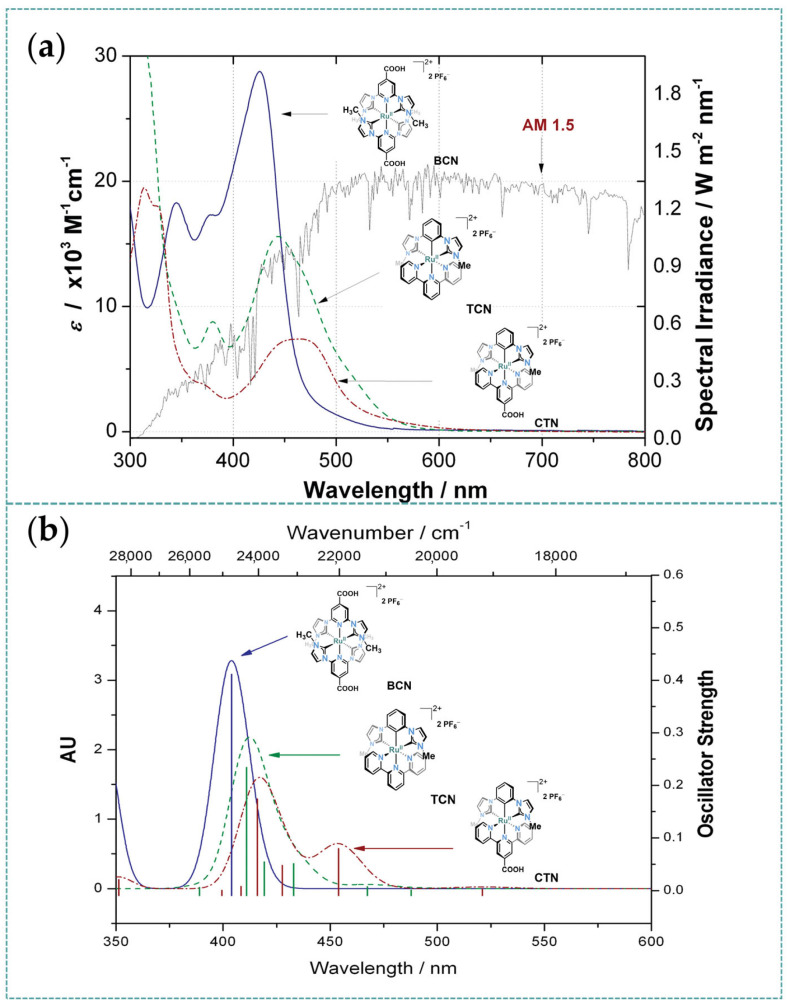
(**a**) Electronic absorption spectra of **BCN** (solid blue), **TCN** (dashed green), and **CTN** (dash-dotted red) complexes. Solar spectral irradiance (AM 1.5) is shown as a gray line. Experimental conditions: solvent = CH_3_CN, temperature = 23 °C; (**b**) simulated electronic absorption spectra of **BCN**, **TCN**, and **CTN** via TD-DFT method. The same basis sets and effective core potentials were used for the ground-state calculations. To consider the solvation effect, CPCM implemented in the Gaussian 03 program package was used. Vertical lines represent the oscillator strengths of **BCN** (blue), **TCN** (green), and **CTN** (red). The peak shape of each individual transition was assumed to be a Gaussian curve with a full width at half-maximum (FWHM) of 1000 cm^−1^.

The electronic absorption spectra of heteroleptic Ru(II) complexes bearing CNC-pincer ligands (**BCN**, **TCN**, and **CTN)** have been reported by the same group (Figure 18). A red shift in the absorption profiles was observed from **BCN** to **TCN** to **CTN**, with absorption maxima at 430, 448, and 463 nm, respectively (Figure 18a). These shifts represent an incremental change from the primitive **[Ru^II^(bip)_2_]^2+^** complex by 2922, 3857, and 4580 cm^−1^, respectively. **CTN** exhibits a broad absorption profile in the lowest-energy region, indicative of multiple overlapping absorption features. Similarly, **TCN** exhibits additional features at 520 and 470 nm, extending beyond its primary absorption maximum at 448 nm. These nuances suggest that the absorption maxima for **TCN** and **CTN** are not merely due to simple HOMO-LUMO transitions. In contrast, **BCN** displays a distinct Gaussian distribution of absorption centered at 430 nm. Despite having similar structural frameworks, these complexes show markedly different absorption intensities. Specifically, **BCN** exhibits a strong extinction coefficient of 28,100 M^−1^ cm^−1^, while **TCN** and **CTN** have weaker coefficients of 15,400 and 7400 M^−1^ cm^−1^, respectively. As supported by TD-DFT calculation (Figure 18b), the significantly higher absorptivity of **BCN** than that of **TCN** may be attributed to the stronger absorptivity of carboxylate-modified bipyridyl ligands compared to ordinary tpy ligands [38].

**Figure 19:**
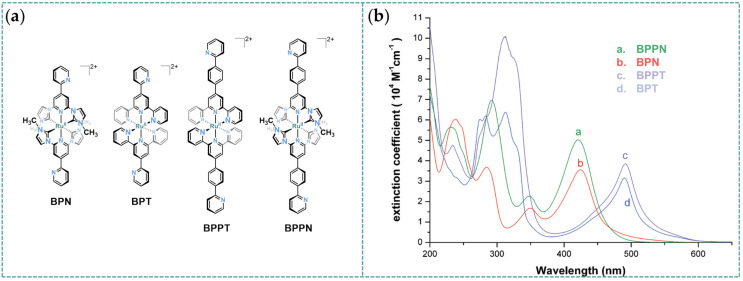
(**a**) Molecular structures of investigated complexes **BPN**, **BPT**, **BPPT**, and **BPPN**; (**b**) electronic absorption spectra of a. **BPPN** (red line), b. **BPN** (violet line), c. **BPPT** (blue line), and d. **BPT** (green line) complexes (2.0 × 10^−5^ M in acetonitrile at 23 °C).

For Ru(II) complexes bearing pyridine-functionalized tpy and CNC-pincer bis-NHC ligands, the absorption profiles of **BPT**, **BPPT**, **BPN**, and **BPPN** in CH_3_CN are shown in Figure 19a. These complexes exhibit strong bands between 200 and 370 nm, associated with π → π* intraligand transitions, while moderate visible bands correspond to ^1^MLCT transitions. Generally, absorption maxima of Ru(II) complexes bearing the NHC pincer rise in a higher-energy region than those of Ru(II)-polypyridyl counterparts, with **BPN** and **BPPN** showing maxima at 424 nm and 425 nm, respectively (Figure 19b). In contrast, the tpy-based **BPT** and **BPPT** exhibit remarkably red-shifted absorption signals centered at 491 nm and 494 nm, respectively. Notably, **BPN** and **BPPN** exhibit stronger molar absorption coefficients than **BPT** and **BPPT**. At ambient temperature, **BPN** and **BPPN** exhibit blue-shifted emission maxima and higher emission intensities compared to **BPT** and **BPPT**. Quantum yields for **BPN** and **BPPN** were determined to be 8.1 × 10^−4^ and 2.5 × 10^−3^, respectively, while those for **BPT** and **BPPT** could not be determined due to their weaker emission intensity. Considering the excited-state lifetimes in O_2_-free CH_3_CN, **BPPT** has a predominant emission lifetime of 0.9 ns (80%) and a minor lifetime of 4.8 ns (20%), while **BPPN** follows a similar pattern with a major decay time of 2.2 ns (85%) and a minor one at 13 ns (15%). On the other hand, **BPT** and **BPN** give major excited-state lifetimes of 0.8 ns (67%) and 2.5 ns (83%), respectively, with longer decay times of 4.6 ns (33%) for BPT and 13 ns (17%) for **BPN** [39].

**Figure 20:**
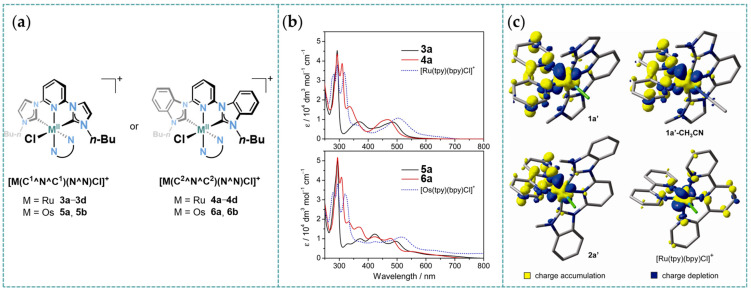
(**a**) Molecular structures of investigated complexes **[Ru^II^(C^N^C)(N^N)L]^n+^** and **[Os^II^(C^N^C)(N^N)Cl]^+^**; (**b**) UV-visible absorption spectra of **[M^II^(C^N^C)(bpy)Cl]^+^** and **[M^II^(tpy)(bpy)Cl]^+^** in CH_3_CN at 298 K; (**c**) electronic difference density plots for **3a′**, **4a′**, **3a′-CH_3_CN**, and **[Ru^II^(tpy)(bpy)Cl]^+^** of the lowest-energy excited states.

**Figure 21:**
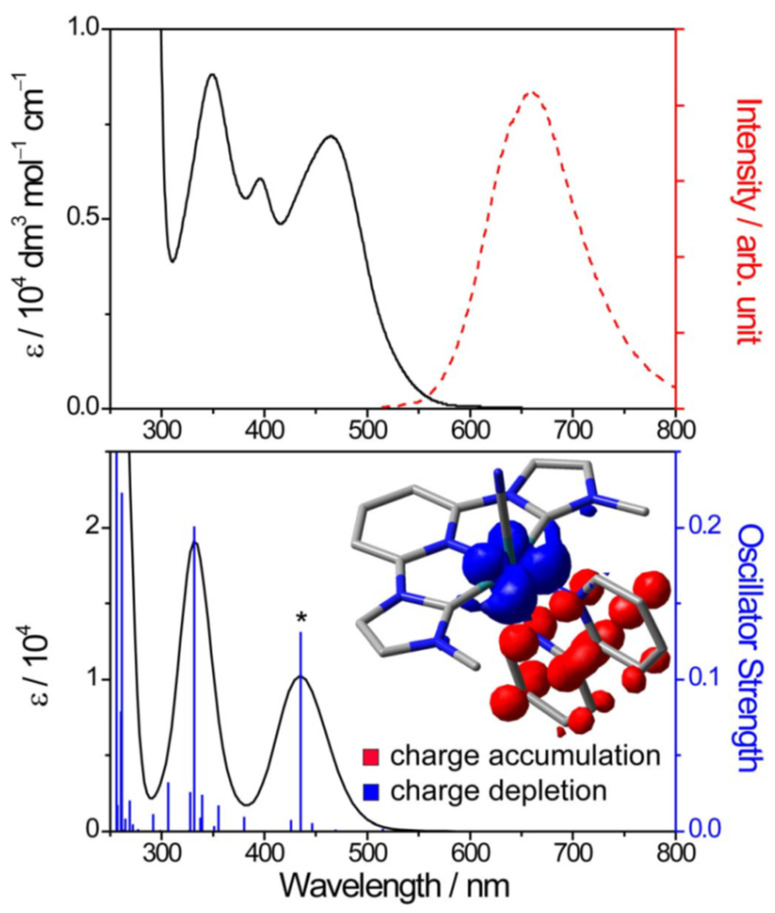
(**top**) Absorption (solid line) and emission (dash line, λ_ex_ = 450 nm) spectra of **[Ru^II^(C^N^C)(bpy)(CN)]^+^** in degassed CH_3_CN at 298 K. (**bottom**) TD-DFT calculated absorption spectrum for model **[Ru^II^(C^N^C)(bpy)(CN)]^+^** in CH_3_CN. Excitation energies and oscillator strengths are indicated by the blue vertical lines; the spectrum (in black) is convoluted with a Gaussian function having a full width at half-maximum of 3000 cm^−1^. The inset shows the electronic difference density plot for the model **[Ru^II^(C^N^C)(bpy)(CN)]^+^** at the vertical transition marked with * (isodensity value = 0.004 au).

Beyond bis-tridentate systems, Wong and co-workers investigated the photophysical properties of **[Ru^II^(C^N^C)(N^N)L]^n+^** (L = Cl^−^, n = 1; L = CH_3_CN, *t*-BuNC, n = 2) and **[Os^II^(C^N^C)(N^N)Cl]^+^**, using NHC-based pincer ligands (C^1^^N^C^1^ and C^2^^N^C^2^) (Figure 20a). UV-visible spectra of these two series of complexes revealed high-energy bands (λ ≤ 320 nm, ε_max_ ≥ 2 × 10^4^ M^−1^ cm^−1^) and lower-energy transitions (λ ≥ 320 nm, ε_max_ ≈ 1 × 10^4^ M^−1^ cm^−1^), with the lowest-energy absorption assigned to dπ(Ru^II^/Os^II^) → π*(N^N) MLCT rather than the expected dπ(Ru^II^/Os^II^) → π*(C^N^C) transition (Figure 20b). Notably, C^2^^N^C^2^ complexes exhibited a blue shift of 749–803 cm^−1^ compared to C^1^^N^C^1^, reflecting the weaker donor strength of benzimidazol-2-ylidene. It is noteworthy that the absorption beyond 500 nm of Os(II) complexes was attributed to spin-forbidden triplet charge-transfer transitions enhanced by strong spin-orbit coupling. TD-DFT calculations showed that the lowest LUMOs are primarily localized on the N^N ligands (26.4–50.7% contribution), with significant contributions from the C^N^C ligands (44.8–78.9%) (Figure 20c). These findings contrast with traditional tpy-based complexes, highlighting the role of ligand architecture in modulating MLCT directionality and offering insights for designing advanced photoactive materials [17]. Amongst these complexes, **[Ru^II^(C^N^C)(bpy)(CN)]^+^** was found to be photoluminescent. In degassed CH_3_CN, the complex exhibited a quantum yield of 8.23 × 10^−3^ (λ_em_ = 659 nm) (Figure 21, top) and an extended lifetime of 1.71 μs, nearly an order of magnitude improvement over Cl^−^ or CH_3_CN-ligated systems. This enhancement is attributed to the strong ligand field of the cyanide ligand, which suppresses non-radiative decay. TD-DFT-derived electronic density difference plots confirmed excited-state electron transfer from the Ru^II^ *d*-orbitals to the bpy ligand (Figure 21, bottom). This work provides a foundation for the interplay between aromatic diimines and NHC pincers in modulating the electronic structures and spectroscopic properties of the complexes [17,40].

**Figure 22:**
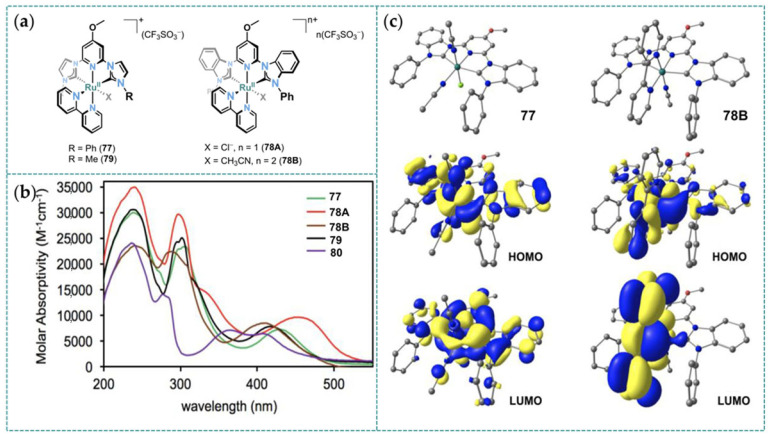
(**a**) Molecular structures of investigated complexes **[Ru^II^(C^N^C)(N^N)(NCCH_3_)]^2+^**; (**b**) electronic spectra for each complex in MeCN; (**c**) frontier molecular orbitals for **77** and **78B** calculated at the SMD-PBE0/BS1 level.

Afterwards, Papish et al. modified the **[Ru^II^(C^N^C)(N^N)(NCCH_3_)]^2+^** system by anchoring the MeO- group on the central pyridine and swapping the substituents (i.e., Me- or Ph-) on the imidazolyl and benzimidazolyl rings (Figure 22a). These complexes feature MLCT transitions (λ_max_ = 410–452 nm) (Figure 22b), with TD-DFT calculations indicating that HOMO mainly localizes on the Ru^II^ *d*-orbitals and NHC ligands, while LUMO is primarily on the bpy ligand (>60%), promoting *d*π(Ru^II^) → π*(bpy) electron transfer (Figure 22c). Phenyl substitution lowers the reduction potential (E_1/2_ = −1.95 V vs. Fc^+^/Fc) and enhances CO_2_ electron transfer capacity (*i*_cat_/*i*_p_ = 2.0–3.7; *i*_cat_ and *i*_p_ correspond to peak currents observed under CO_2_ and N_2_). The weakly coordinated CH_3_CN ligand (Ru–N = 2.094 Å) enhances active site accessibility. This study highlights the impact of π-conjugation, substituent effects, and coordination modes on light harvesting and charge separation, giving insights for developing efficient light-capturing complexes [41].

**Figure 23:**
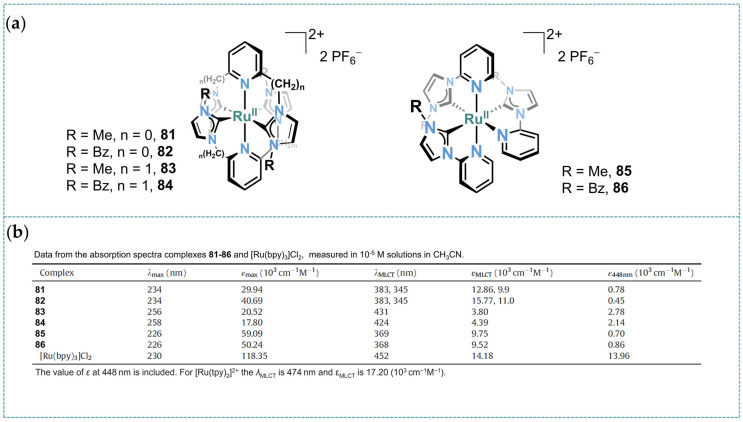
(**a**) Molecular structures of investigated complexes **[Ru^II^(C^N^C)_2_]^2+^** and **[Ru^II^(N^C)_3_]^2+^**; (**b**) data from the absorption spectra of complexes **81**-**86** and **[Ru^II^(bpy)_3_]Cl_2_**, measured in 10^−5^ M solutions in CH_3_CN.

In the same year, Jalón and co-workers reported a series of homoleptic Ru(II) complexes, including **[Ru^II^(C^N^C)_2_]^2+^** and **[Ru^II^(N^C)_3_]^2+^** derivatives with pyridine-imidazole hybrid ligands (Figure 23a), to investigate their photophysical properties and photocatalytic hydrogen evolution reaction (HER) performance. Key insights were gained regarding ligand architecture and substituent effects demonstrated by important tabulated photophysical data (Figure 23b). For **[Ru^II^(C^N^C)_2_]^2+^**, benzimidazole-based analogues (e.g., **82**, **84**) exhibited red-shifted MLCT absorption bands compared to the imidazole counterpart (e.g., **82**: λ_max_ = 452 nm vs. **81**: λ_max_ = 438 nm) due to extended π-conjugation from phenyl groups, enhancing visible-light harvesting. In contrast, analogues bearing methylene-bridged ligands showed blue-shifted MLCT transitions (431 nm for **83** and 424 nm for **84**), attributable to the destabilized LUMOs by disrupted conjugation. It was found that only methyl- (**81**) and benzyl-substituted (**82**) derivatives emit at room temperature, with broad MLCT emission profiles (λ_max_ centered at 532–528 nm) and large Stokes shifts (~5600 cm^−1^). Benzyl substitution marginally improved quantum yields (Φ_PL_: 0.18% vs. 0.16%) and extended excited-state lifetimes (τ: 1.7 μs vs. 1.5 μs), suggesting steric suppression of non-radiative decay. Phenyl groups enhanced π-delocalization to stabilize charge-separated states, while methylene bridges compromised conjugation. In contrast, bidentate ligand systems (**85** and **86**) exhibited remarkably blue-shifted absorption (λ_MLCT_ ~369 nm) due to the raised LUMO energy levels imposed by geometric constraints. This study links MLCT properties (absorption range, excited-state lifetime, and charge separation efficiency) to ligand modifications, providing a strategy for designing efficient Ru-based photosensitizers through controlled π-conjugation and steric modulation [42].

**Figure 24:**
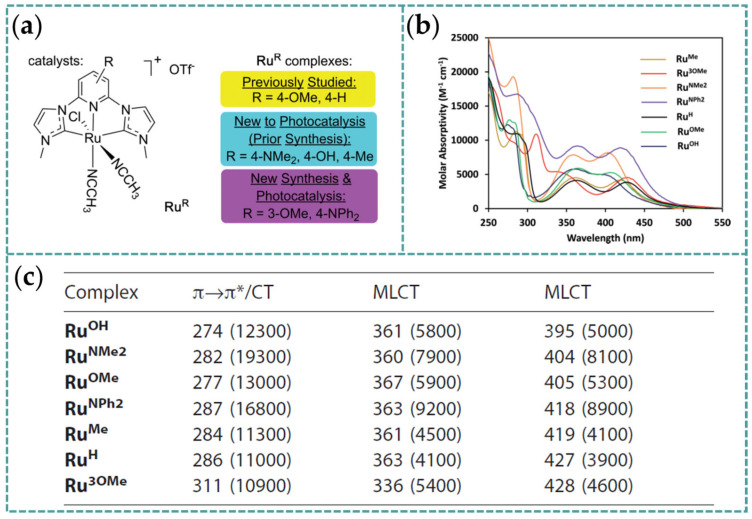
(**a**) Molecular structures of investigated complexes **[Ru^II^(CNC)(CH_3_CN)_2_Cl]OTf**; (**b**) experimental absorption spectra of each complex in CH_3_CN; (**c**) electronic spectral features for the studied **[Ru^II^(CNC)(CH_3_CN)_2_Cl]OTf**. These are sorted by the energy of the lowest energy MLCT band. Assignments are based on experimental and computational data.

One year later, Papish and co-workers studied the photophysical properties of seven Ru(II) CNC-pincer complexes featuring varied substituents (**Ru^OH^**, **Ru^NMe2^**, **Ru^OMe^**, **Ru^NPh2^**, **Ru^Me^**, **Ru^H^**, **Ru^3OMe^**) (Figure 24a). Key findings (detailed tabulated data, see Figure 24c) include that *para*-substituted π-donors (e.g., **Ru^OMe^**, **Ru^NMe2^**) donate electrons to the pyridyl ring, lowering LUMO energy and red-shifting MLCT absorption (λ_abs_ = 405 nm, ε_max_ = 5300 M^−1^ cm^−1^), while *meta*-substituents (e.g., **Ru^3OMe^**) lack resonance and lead to blue-shifted absorption (λ_max_ = 428 nm) (Figure 24b). Strong π-donors in **Ru^NPh2^** enhance MLCT absorption centered at 418 nm (ε_max_ = 8900 M^−1^ cm^−1^) better than σ-donors (e.g., **Ru^Me^**; λ_abs_ = 419 nm, ε_max_ = 4900 M^−1^ cm^−1^). On the other hand, **Ru^OH^** exhibits a blue-shifted absorption peak centered at 395 nm. **Ru^Me^** and **Ru^NPh2^** exhibit MLCT emission (λ_em_ = 528–532 nm) with large Stokes shifts (~5600 cm^−1^), indicating charge-separated excited states. This work gives an overview of the correlation between electronic character and the position of substituents. *Para*-π-donors optimize MLCT transitions, while σ-donors balance absorption and stability. Oxidatively labile groups (e.g., –NMe_2_, –OH) induce decomposition, suggesting their exclusion in catalyst design. This work provides a reference for the rational design of high-performance photosensitizers through the manipulation of substituent effects [43].

**Figure 25:**
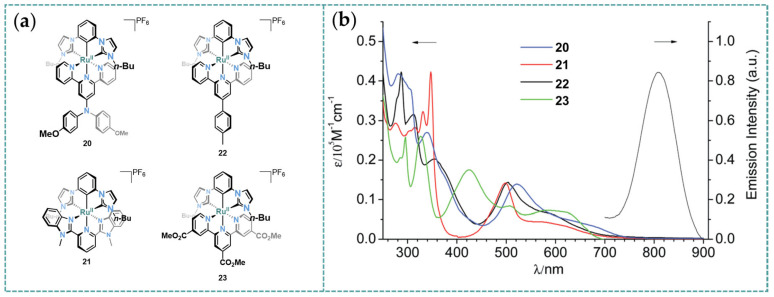
(**a**) Molecular structures of investigated cyclometallated Ru(II) complexes **20**-**23**; (**b**) UV/vis absorption and emission spectra of **20**-**23** in CH_3_CN.

Moving from CNC-pincer bis-NHC complexes, Zhong et al. prepared cyclometallated Ru(II) complexes (**20**–**23**) featuring CCC-pincer bis-NHC ligands (Figure 25a), exploring ligand engineering to modulate their photophysical and redox properties. The strong σ-donor nature of the NHC ligands lowers Ru^II/III^ oxidation potentials, as demonstrated by complex **22** (+0.51 V vs. Ag/AgCl), 50 mV lower than the pyridine-based **[Ru^II^(dpb)(tpy)]^+^**. The influence of ligand substituents on the photophysical properties has been studied (Figure 25b). *Para*-π-donors (e.g., 4-di-*p*-anisylamino) redshift MLCT absorption (λ_abs_ = 520 nm, ε_max_ ≈ 10^4^ M^−1^ cm^−1^) by stabilizing LUMO, while *meta*-substituents (e.g., 3-OMe) result in blue shifts (λ_abs_ = 428 nm). Electron-deficient ligands, such as Me_3_cbtpy, shift the Ru^II/III^ potential to +0.75 V and broaden MLCT absorption (400–700 nm). TD-DFT calculations reveal that MLCT transitions originate from the metal-to-non-cyclometallating ligand charge transfer due to the electron-donating CCC-pincer framework. Complex **22** shows weak emission centered at 808 nm (Φ < 0.01%) due to non-radiative decay, while complexes **20**, **21**, and **23** display negligible room-temperature photoluminescence. This work establishes the first CCC-pincer bis-NHC Ru platform, providing control over MLCT directionality and redox potentials, with implications for optoelectronic materials in dye-sensitized solar cells [23].

**Figure 26:**
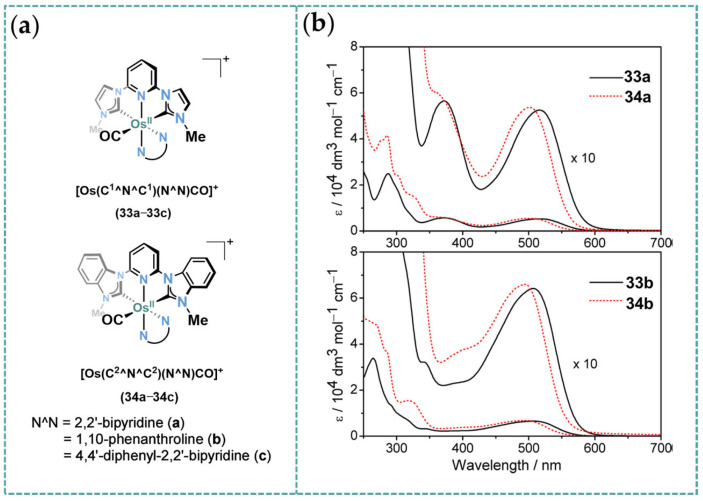
(**a**) Molecular structures of investigated complexes **[Os^II^(C^C^C)(N^N)(CO)]^+^**; (**b**) UV-visible absorption spectra of selected complexes in CH_3_CN at 298 K.

Considering the CCC-pincer bis-NHC chelates, our group has reported the first work incorporating this ligand into the Os(II) system, investigating its photophysical nature, electronic structure, and photoluminescent properties. The Os(II) complexes are in the form of **[Os^II^(C^C^C)(N^N)(CO)]^+^**, where C^C^C are the 1,3-bis(1-methylimidazolin-2-ylidene)phenyl anion or benzimidazolinyl analogue and N^N are bpy, phen, or Ph_2_bpy (Figure 26a). Spectroscopic and TD-DFT studies show that the lowest-energy absorption bands (λ_abs_ = 493–536 nm, ε_max_ = 5–10 × 10^3^ M^−1^ cm^−1^) arise from *d*π(Os^II^) → π*(N^N) MLCT transitions (Figure 26b). The C^C^C ligands influence the hybrid [Os + C^C^C] frontier orbitals via significant contributions to HOMO (Os *d*-orbitals and C^C^C σ-donation) and LUMO (dominated by N^N π* orbitals). All complexes exhibit red emission (λ_em_ = 674–731 nm) with low quantum yields (Φ = 10^−4^–10^−2^) but extended lifetimes (τ = 1–6 μs), much longer than classical **[Os^II^(bpy)_3_]^2+^** (60 ns). Transient absorption and spectroelectrochemical measurements confirm the MLCT nature of the emissive states, while C^C^C ligands enhance excited-state stability by reducing non-radiative decay. This study highlights the role of NHC ligands in optimizing the photophysics of the Os(II) luminophore, offering a strategy for designing long-lived red-emitting materials for optoelectronic applications [26].

**Figure 27:**
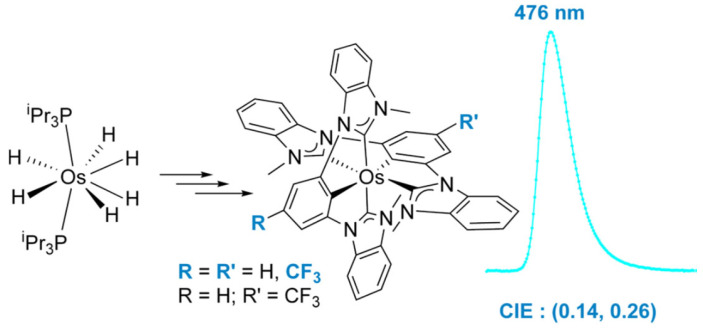
General scheme showing the synthetic access to blue-emitting **[Os^II^(C_NHC_C_aryl_C_NHC_)_2_]** and one of the strong sky-blue emission signals.

Considering the homoleptic **[Os^II^(C_NHC_C_aryl_C_NHC_)_2_]** and heteroleptic **[Os^II^(C_NHC_C_aryl_C_NHC_)(C_NHC_C_aryl’_C_NHC_)]** complexes prepared by Esteruelas and Xia, these complexes have been studied for their photophysical and photoluminescent properties. These compounds exhibited efficient blue–green emission in the solid state (475–578 nm, Figure 27), with the homoleptic complex **[Os^II^(C_NHC_C_aryl_C_NHC_)_2_]** achieving a remarkably high photoluminescence quantum yield of 0.62 and an emission lifetime of 28 μs, attributed to joint contributions from MLCT and ligand-centered π → π* transitions. Solution-phase studies revealed blue-shifted emission at 77 K, indicative of enhanced rigidity in the excited state [27].

### 3.2. Photophysical Properties of Ir-CCC Complexes

In 2013, De Cola and co-workers studied the photophysics of two cationic near-UV emitters based on bis-pincer Ir(III) carbene complexes, **[Ir^III^(*^n^*^Bu^C_NHC_^Me^CC_NHC_*^n^*^Bu^)_2_]I** and **[Ir^III^(*^n^*^Bu^C_NHC_^Me^CC_NHC_*^n^*^Bu^)_2_]PF_6_**. In CH_3_CN, both complexes exhibited strong absorption bands at 270 nm (ε_max_ = 1.1–1.5 × 10^4^ M^−1^ cm^−1^), 290 nm, and 320 nm, attributed to mixed ^1^MLCT/^1^LC transitions. Under O_2_-free conditions, they emitted intensely at 384 nm and 406 nm (Φ = 0.41 and 0.38). In the solid state, low-energy emission at 500 nm was observed for both, but the ratio of high- to low-energy emission varied with the counterion. The **[Ir^III*n*Bu^(C_NHC_^Me^CC_NHC_)_2_]I** crystals showed a broad peak centered at 500 nm (Φ = 0.12) with shortened lifetimes (1.0–4.7 μs), while the PF_6_^−^ complex exhibited dual emission (384/406 nm + 500 nm, Φ = 0.20). The low-energy emission was linked to aggregation-induced trapping sites, where weaker C–H···I interactions in the iodide complex promoted defects, while stronger C–H···F interactions in the PF_6_^−^ analogue suppressed aggregation. This study highlights the importance of counterions in tuning solid-state luminescence, offering insights for designing near-UV emitters for electroluminescent devices [44].

**Figure 28:**
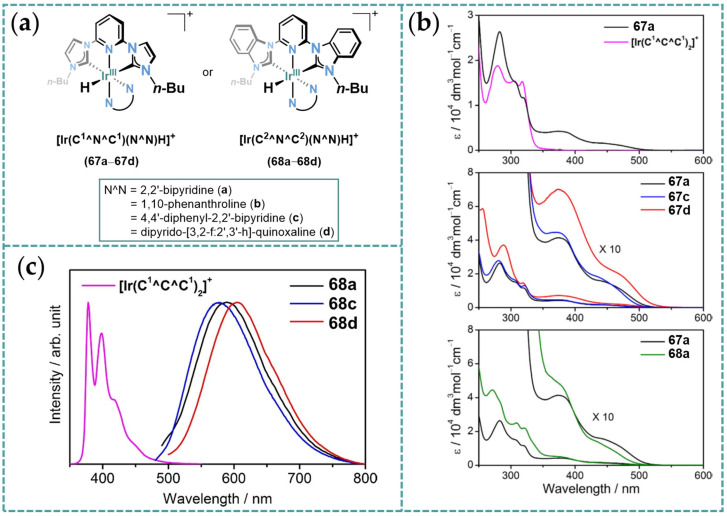
(**a**) Molecular structures of investigated complexes **[Ir^III^(C^C^C)(N^N)(H)]^+^**; (**b**) UV-visible absorption spectra of selected complexes in CH_3_CN at 298 K; (**c**) emission spectra for **68a**, **68c**, **68d**, and **[Ir^III^(C^1^^C^C^1^)_2_]^+^** (λ_ex_ = 420 nm for **67**-**68**, 340 nm for **[Ir^III^(C^1^^C^C^1^)_2_]^+^**).

Two years later, our group expanded the molecular system bearing both CCC-pincer bis-NHC ligands and aromatic diimines to an Ir(III)-based system, **[Ir^III^(C^C^C)(N^N)(H)]^+^** (Figure 28a). The complexes exhibited low-intensity absorption bands at 340–530 nm (ε_max_ = 10^3^ M^−1^ cm^−1^) (Figure 28b), attributed to *d*π(Ir^III^) → π*(N^N) ^3^MLCT transitions, with the C^C^C ligands modulating transition energies by enriching the electron density of the *d*π(Ir^III^) orbital. Complex **67a** (C^1^^C^C^1^) showed λ_max_ at 374 nm, while that of **68a** (C^2^^C^C^2^) was red-shifted to 422 nm. Emission spectra revealed yellow emission (λ_em_ = 553–604 nm) (Figure 28c), with quantum yields (Φ = 10^−3^–10^−1^) and lifetimes (τ = 10–790 ns) varying by ligand. Notably, complex **67b** with the phen ligand achieved Φ = 0.119 and τ = 790 ns, outperforming **68d** with dpq (Φ = 0.0116), highlighting the electron-donating effect of ligand moieties in reducing non-radiative decay. TD-DFT calculations revealed the ^3^MLCT excited state, with C^C^C contributing 27–69% to HOMO and N^N dominating LUMO (93%). This study demonstrates how NHC-based pincer ligands optimize MLCT emission through *d*π orbital engineering, offering insights for designing efficient yellow-emitting Ir(III) complexes [35].

**Figure 29:**
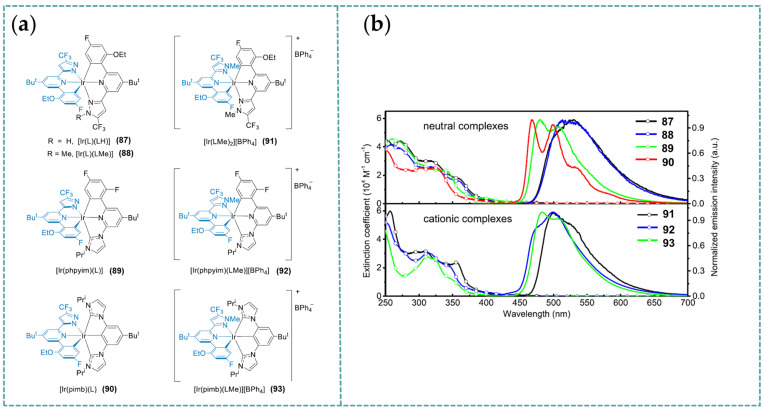
(**a**) Molecular structures of investigated bis-tridentate Ir(III) complexes **87**-**93**; (**b**) absorption and emission spectra for neutral Ir(III) complexes **87**-**90** and cationic complexes **91**-**93** recorded in degassed CH_2_Cl_2_ solution at ambient temperature, respectively.

Chou and Chi et al. contributed significantly to the investigation of photophysical properties of bis-tridentate Ir(III) complexes (Figure 29a) for OLED applications, exploring how ligand structures influence luminescence. Complexes with bis(imidazolylidene)benzene and 2-(5-trifluoromethylpyrazolyl)-6-(2,4-difluorophenyl)pyridine showed efficient sky-blue emission (λ_em_ = 463–472 nm, Φ = 75–95%) with intraligand π → π* transitions and lifetimes of 2.58–4.67 μs (Figure 29b). Replacing phenyl groups with phenoxy moieties and adding NMe_2_ donors (e.g., 6-pyrazolyl-2-phenoxypyridine) shifted emission to the deep-blue region (λ_em_ = 468 nm, CIE (0.14, 0.15)), achieving a 100% quantum yield in the solid state and a shorter radiative lifetime (0.67 μs), with an external quantum efficiency (EQE) of 16.8%. Substituents like *meta-*/*para-*CF_3_ modulated MLCT/LLCT contributions, improving emission efficiency. Alternatively, dual methyl/CF_3_ modifications increased stability and shortened lifetimes [45,46,47,48]. Afterwards, they also explored how ligand design (carbene content and charge states) in bis-tridentate Ir(III) complexes affects photophysical properties. Neutral complex **[Ir(pimb)(L)]** with imidazolylidene carbenes exhibited excellent blue emission (Φ = 100%, λ_em_ = 469/531 nm, τ = 4.06 μs). In contrast, the cationic complex **[Ir(pimb)(LMe)][BPh_4_]** showed a reduced quantum yield (10%) due to a narrow energy gap (2.66 kcal mol^−1^) between triplet metal-centered ^3^MC (*dd*) and ^3^MLCT/ππ* states, leading to higher non-radiative decay (6.0 × 10^6^ s^−1^). Theoretical calculations have shown that a stronger ligand field strength widens the energy gap, thereby suppressing non-radiative transitions. OLED devices with **[Ir(phpyim)(L)]** and **[Ir(pimb)(L)]** achieved EQEs of 16.7% and 14.6%, demonstrating the potential of these complexes as efficient blue phosphorescent materials. These works highlight the role of ligand electronic structure and steric effects in emission mechanisms and device performance, offering guidelines for designing high-efficiency deep-blue phosphorescent materials [49].

### 3.3. Photophysical Property of Rh-^R^CNC^R^ Complexes

**Figure 30:**
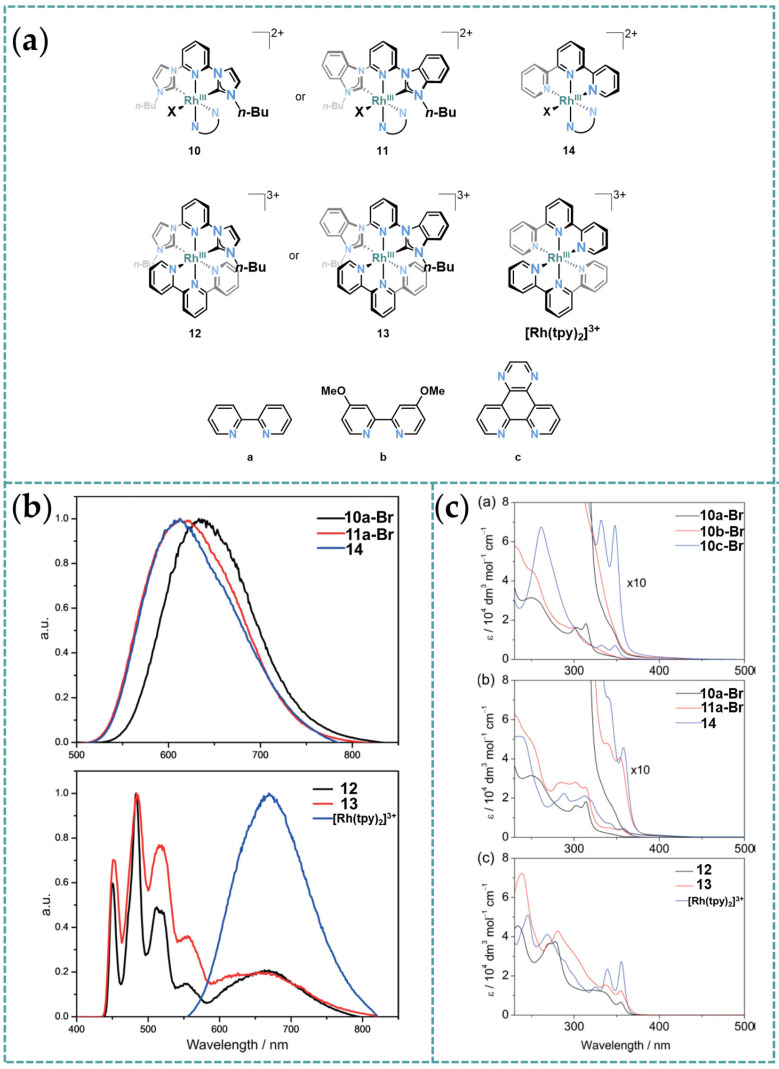
(**a**) Molecular structures of investigated Rh(III) complexes, **10**-**14**; (**b**) emission spectra for **10**-**14**, **[Rh^III^(tpy)_2_]^3+^** in *n*-butyronitrile at 77 K; (**c**) UV-Vis absorption spectra for **10**-**14**, **[Rh^III^(tpy)_2_]^3+^** in CH_3_CN at 298 K.

Pinpointing the analogue of Ir, our group has studied the photophysical properties of Rh(III) complexes equipped with CNC-pincer bis-NHC ligands, including 2,6-bis(1-butylimidazol-2-ylidene)pyridine and 2,6-bis(3-butylbenzimidazol-2-ylidene)pyridine, coordinated with bpy/tpy ligands (Figure 30a). At 77 K, complexes with bpy ligands (e.g., **[Rh^III^(C^1^^N^C^1^)(bpy)Br]^2+^**) exhibited broad phosphorescent emission at 610–660 nm (lifetimes of 31–54 μs), attributed to ^3^MC excited states (Figure 30b). On the other hand, tpy complexes (e.g., [**Rh^III^(C^1^^N^C^1^)(tpy)]^3+^**) displayed mixed emissions, with structured triplet ligand-centered (^3^LC) transitions at 450–560 nm and ^3^MC emissions at 660–670 nm (lifetimes of 68–75 μs). All the studied Rh(III) complexes exhibited a high-energy absorption tail extending to around 400 nm (Figure 30c). TD-DFT calculations revealed that low-energy transitions involved *d*π(Rh^III^) → π*(bpy/tpy) MLCT and LC transitions. Comparison with **[Rh^III^(tpy)_2_]^3+^** (which exhibits ^3^MC emission) revealed that the strong σ-donor effect of NHC ligands raises the energy of ^3^MC states, reducing non-radiative decay and enhancing ^3^LC luminescence. This work highlights the role of NHC pincer ligands in stabilizing emissive states, offering a strategy for controlling excited-state energy levels in Rh(III) complexes [20].

## 4. Application of *N*-Heterocyclic Carbene Metal Complex

### 4.1. Photocatalytic Carbon Dioxide Reduction

In the pursuit of effective artificial photosynthesis, the visible-light-driven photocatalytic CO_2_ reduction reaction (CO_2_RR) is a critical reaction. The key challenge in this process is developing photocatalysts that not only efficiently and selectively reduce CO_2_ but also remain intact over a long operational period. Homogeneous catalysts, especially those composed of transition metals, have emerged as rising stars for both photocatalytic and electro-catalytic applications in CO_2_ reduction.

**Figure 31:**
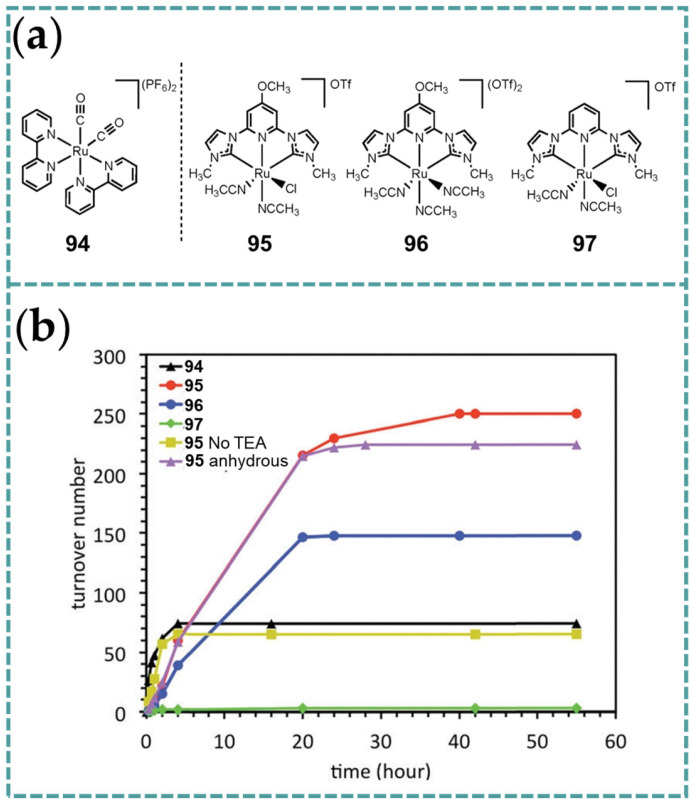
(**a**) Ru(II) benchmark catalyst **94** and Ru(II) CNC-pincer complexes **95**–**97**; (**b**) TONs for CO production for complexes **94**-**97** upon irradiating with a solar simulator. Each data point is from a duplicate experiment.

In 2017, Papish et al. introduced a novel Ru(II) complex catalyst with substituted pyridine-bridged bis-NHC pincer ligands (i.e., **[Ru^II^(CNC)(NCCH_3_)_3−n_Cl_n_]^(2−n)+^**, n = 0 or 1, **95**–**97**) for efficient photocatalytic CO_2_RR to CO (Figure 31a). Under optimized conditions with ***fac-*[Ir(ppy)_3_]** as the photosensitizer (PS), 1,3-dimethyl-2-phenyl-2,3-dihydro-1H-benzoimidazole (BIH) as the scavenger, and triethylamine (TEA) in CH_3_CN, catalyst **95** achieved a CO turnover number (TON) of 250 and 100% selectivity over 40 h (Figure 31b). This performance surpassed the benchmark catalyst **[Ru(bpy)_2_(CO)_2_]^2+^**, **94**, which deactivated after 4 h operation. A key insight was the importance of the *para*-methoxy substituent on the pyridine ring; catalyst **95** (**Ru^OMe^**) outperformed its unsubstituted analogue **97** (**Ru^H^**; TON = 3) significantly. This work marked the first successful use of Ru(II) complexes bearing a substituted pyridine-bridged bis-NHC pincer in CO_2_ photoreduction, highlighting a new approach to ligand design by incorporating electron-donating groups to enhance catalytic activity and stability [50].

Subsequently, the same group evaluated factors affecting photocatalytic CO_2_RR, including solvents, e^−^/H^+^ sources, PS, and catalysts. They found that environmental factors, particularly the choice of H^+^ sources and photosensitizers, could significantly influence product distribution, sometimes more than the catalyst design itself. For example, the catalyst **[Ru^II^(CNC)(NCCH_3_)_2_Cl]^+^**, typically CO-selective, switched to predominantly producing formate (HCOO^−^, 90% selectivity) after switching the PS from ***fac-*[Ir(ppy)_3_]** to **[Ru^II^(bpy)_3_]^2+^**, while **[Ru^II^(bpy)_2_(CO)_2_]^2+^** generated exclusively CO after changing the PS and scavenger. Furthermore, self-sensitized **[Ru^II^(CNC)(bpy)(NCCH_3_)]^2+^** was used for photocatalytic CO_2_RR without the use of external PS. By using benzimidazole-derived pyridine-bridged bis-NHC pincer and bpy ligands, this catalyst exhibits enhanced visible-light absorption and catalytic activity through MLCT. It achieved an initial turnover frequency (TOF) of 8.3 h^−1^ for CO, along with a total TON of 33,000 at a 1 nM concentration under PS-free conditions [41,51].

**Figure 32:**
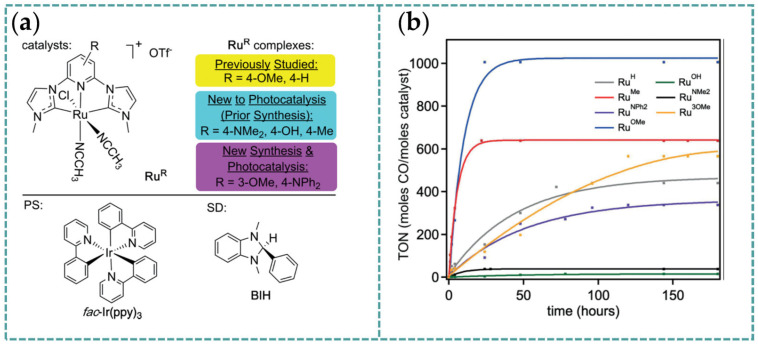
(**a**) Structures of the **[Ru^II^(CNC)(CH_3_CN)_2_Cl]OTf** catalysts, PS, and sacrificial donor used for CO_2_RR; (**b**) TON vs. time plot for the photocatalytic CO_2_RR with all the catalysts.

In 2020, the same group investigated the effect of R substituents on the pyridyl moiety of the CNC-pincer bis-NHC ligand to unveil the structure–activity correlations in the previously studied **[Ru^II^(CNC)(NCCH_3_)_2_Cl]^+^** complexes [43]. These Ru(II) complexes (concentration of 100 μM) were tested for photocatalytic CO_2_RR using ***fac-*[Ir(ppy)_3_]** as PS and a mixture of BIH and TEA as scavengers (Figure 32a). To avoid scavenger-limited catalysis (e.g., the maximum solubility of BIH in CH_3_CN is 10 mM), this study was performed at lower catalyst concentrations compared to previously studied **Ru^OMe^** and **Ru^H^** catalysts. It was found that the TON values follow the sequence of **Ru^OMe^** > **Ru^Me^** > **Ru^3OMe^** > **Ru^H^** > **Ru^NPh2^** >> **Ru^NMe2^** > **Ru^OH^** (Figure 32b), with **Ru^OMe^** achieving a TON of 1006 and a TOF of 174 h^−1^ (Table 1). Notably, substituting a 4-OMe group with a 4-Me group significantly lowered both durability and turnover rate.

Noteworthily, this study highlighted the importance of the position of the -OMe group, as catalysts with 4-OMe outperformed those with 3-OMe. Control experiments confirmed that CO production was linked to the catalytic system. However, the performance of **Ru^H^** varied due to the occasional formation of white precipitates during catalysis. Additionally, the -NPh_2_ donor in **Ru^NPh2^** led to lower TON and TOF than other substituents, raising concerns about potential catalyst decomposition through a one-electron oxidation pathway. The least effective catalysts were **Ru^NMe2^** and **Ru^OH^**. Overall, these findings suggest that stable π-donor groups on catalyst ligands are crucial for effective photocatalytic CO_2_RR and provide insights for future catalyst design.

### 4.2. Bio-Imaging Study

**Figure 33:**
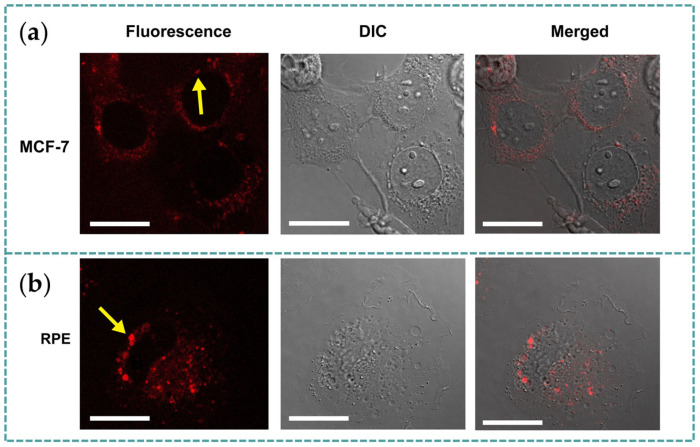
Luminescent images, differential interference contrast (DIC) images, and the overlay of these images (merged) of the live MCF-7 cells (**a**) and RPE cells (**b**) incubated with **[Ru^II^(CNC)(bpy)(CN)]^+^**. Two examples of the mapping of luminescent signals to vesicles present in the cytoplasm are indicated by arrows. Scale bars represent 25 μm.

Photoluminescent Ru^II^-polypyridyl complexes have long been utilized as biological probes or biomedicines due to their compatibility with cell matrices, specificity to certain organelles and strong intrinsic photoluminescence. In 2014, our group developed **[Ru^II^(CNC)(bpy)(CN)]^+^**. This strongly emissive complex bearing a stable molecular framework and auxiliary -CN group demonstrated excellent biocompatibility, being non-toxic to human breast carcinoma cells (MCF-7) and non-cancerous retinal pigmented epithelium cells (RPE). It also showed promise as a luminescent probe for tracking endocytosis in these cell types. This complex exhibited notable photoluminescence with an λ_em_ of 659 nm in CH_3_CN and 648 nm in a CH_3_CN/H_2_O mixture. Upon photoexcitation, it exhibited significantly enhanced emission quantum yields (Φ = 8.23 × 10^−3^) and longer lifetimes (1.71 μs) compared to its -Cl, -CH_3_CN, and -*t*-BuNC analogues. These characteristics indicate potential application of **[Ru^II^(CNC)(bpy)(CN)]^+^** in physiological environments. The cytotoxicity assessment using the Prestoblue assay showed **[Ru^II^(CNC)(bpy)(CN)]^+^** to be non-toxic across a range of concentrations (0.1–500 µM), suitable for application as luminescent biological probes. In bio-imaging studies, luminescent signals in RPE cells are localized as discrete puncta under confocal microscopy, indicating vesicular structures (Figure 33). Monitoring of RPE cells revealed that these vesicles moved from peripheral regions toward the nucleus, suggesting internalization through endocytosis and subsequent transport to the Golgi apparatus, with possible subsequent processing or degradation in lysosomal compartments [40].

**Figure 34:**
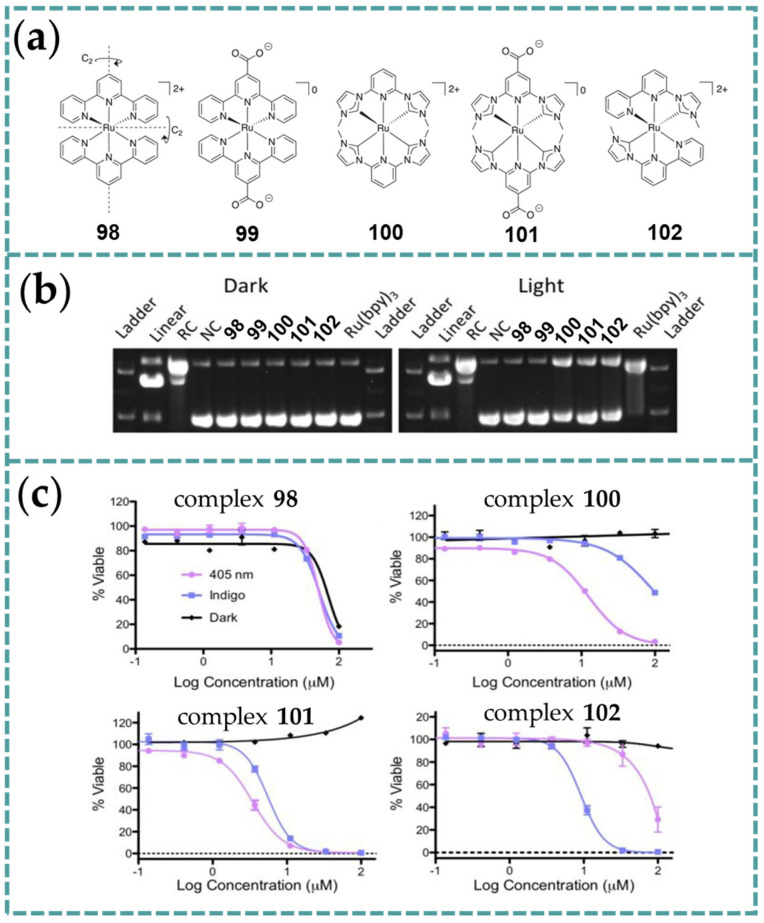
(**a**) Molecular structures of investigated Ru(II) complexes (**98**-**102**); (**b**) agarose gel electrophoresis of pUC19 plasmid (40 μg mL^–1^, 10 mM phosphate buffer, pH 7.4) under dark conditions (left) and after irradiation (right; 470 nm light, 37 J cm^–2^) (all compounds were tested at 500 μM; RC = relaxed circle, NC = no compound); (**c**) cytotoxicity dose responses in HL 60 cells: dark conditions (black diamonds), following irradiation with 405 nm (lavender squares) and 450 nm light (orchid circles) (n = 3).

In 2020, Selegue et al. introduced Ru^II^-NHC complexes (complexes **100**-**102**) to address the problem of short-lived excited states in traditional Ru^II^-polypyridyl systems (e.g., τ = 0.2 ns for **[Ru^II^(tpy)_2_]^2+^**) that limit reactive oxygen species (ROS) generation in photodynamic therapy (PDT) (Figure 34a). By incorporating strong σ-donating NHC ligands, the excited-state lifetimes were extended to 107-529 ns, with complex **100** achieving a 2600-fold improvement over classical systems (e.g., τ = 10^−1^ ns) and surpassing the benchmark **[Ru^II^(bpy)_3_]^2+^** (τ = 376 ns). X-ray crystallography confirmed the *D*_2_*d* symmetry for complexes **100** and **101**, making them achiral and reducing biological interference from stereoisomers, while complex **102** exhibited *C*_2_ symmetry. Photophysical studies showed that complex **101** exhibits intense absorption centered at 415 nm (ε = 26,000 M^−1^ cm^−1^), outperforming traditional tpy systems (e.g., complex **98**: 15,000 M^−1^ cm^−1^, max λ_abs_ = 475 nm). In singlet oxygen (^1^O_2_) production tests, complex **101** generated 50% of the output of **[Ru^II^(bpy)_3_]^2+^** under 450 nm excitation, while tpy systems produced negligible ^1^O_2_ (Figure 34b). Cellular assays confirmed complete dark non-toxicity of the Ru^II^-NHC complexes (IC_50_ > 300 μM), unlike the highly toxic Ru^II^-tpy systems (IC_50_ < 2.3 μM). Upon photoactivation, complex **101** exhibited significant photocytotoxicity, with IC_50_ values of 3.5 μM (λ_ex_ = 405 nm) and 5.6 μM (λ_ex_ = 450 nm), achieving phototoxicity indices (PI) greater than 86 and 54, surpassing 5-aminolevulinic acid (PI > 18) and approaching cisplatin’s potency (IC_50_ = 3.4 μM) (Figure 34c). Mechanistic studies indicated that DNA damage was primarily radical-mediated and that the complexes maintained structural integrity (<7% photodegradation) in various physiological conditions for 72 h. These works advance the design of NHC ligands to create high-performance bis-tridentate Ru(II) complexes for PDT. These complexes are achiral, minimally toxic in the dark, and exhibit micromolar photoactivity, setting a new standard for safe photosensitizers. Future efforts will aim to extend absorption to near-infrared wavelengths and test their in vivo effectiveness [52].

### 4.3. Catalytic Organic Reactions

**Scheme 22:**
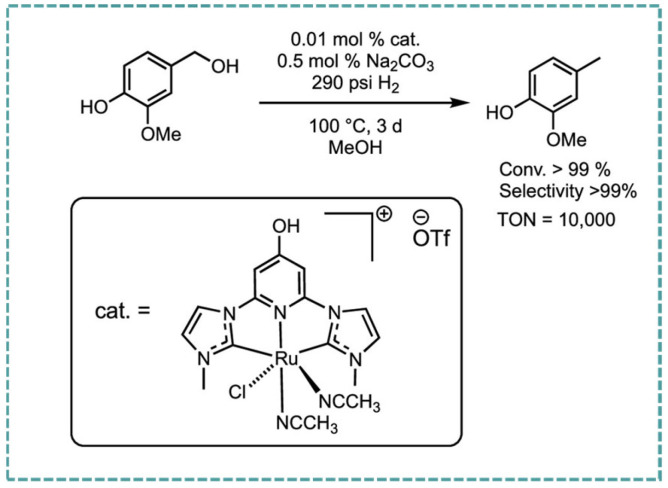
Hydrodeoxygenation of organic substrates with **[Ru^II^(CNC)(NCCH_3_)_2_Cl]^+^**.

**Figure 35:**
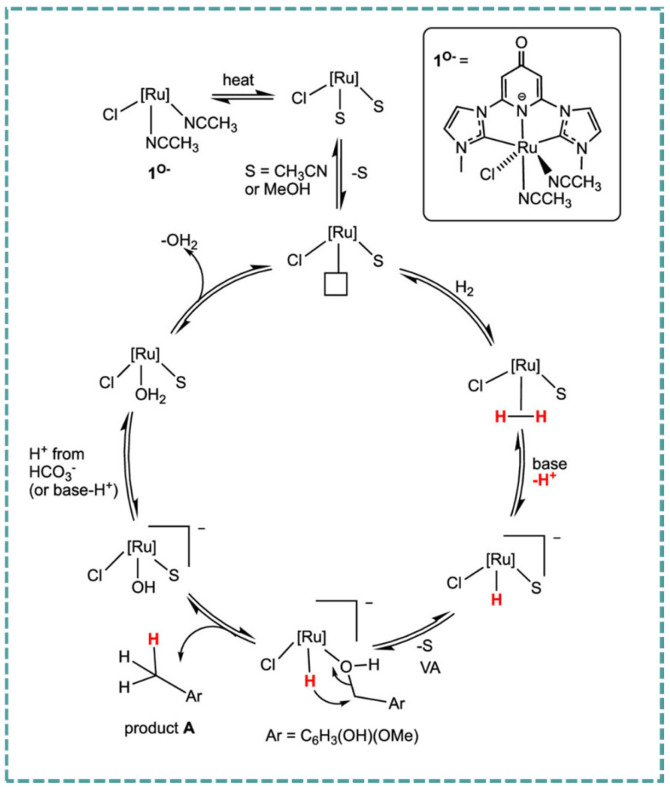
Proposed mechanism for hydrodeoxygenation of vanillyl alcohol with **[Ru^II^(CNC)(NCCH_3_)_2_Cl]^+^**.

Biomass conversion processes, particularly thermal methods such as pyrolysis, gasification, and combustion, play a crucial role in transforming biomass into valuable energy. Pyrolysis generates bio-oil, which suffers from issues such as thermal instability, high viscosity, and a low heating value due to a high oxygen content. Selective deoxygenation has emerged as a solution to reduce the oxygen content. Papish and co-workers adopted electron-rich **[Ru^II^(CNC)(NCCH_3_)_2_Cl]^+^** as a molecular platform to study the catalytic hydrodeoxygenation (HDO) of the biomass molecule, vanillyl alcohol (VA) (Scheme 22). In the catalytic conversion of VA, there are two main products: Product A (creosol), the desired HDO product with a lower oxygen content, and Product B (methyl vanillyl ether), the undesirable one with a retained oxygen content. The addition of an external base, like Na_2_CO_3_, was found to significantly enhance the HDO reaction. A combination of hydroxyl-substituted **[Ru^II^(CNC)(NCCH_3_)_2_Cl]^+^**, **1^OH^**, and 50 mol% Na_2_CO_3_ achieved a 96% yield for Product A in 1 h. Optimization showed that strong bases are detrimental, while weak Na_2_CO_3_ provides optimal conversion and selectivity. Lower catalyst loadings were also evaluated, and it was found that complete conversion can be achieved with just 0.05 mol% catalyst and a high TON of 2000 within an hour. Further lowering the catalyst loading to 0.01 mol% at 100 °C resulted in a TON of 10,000 after a three-day period. The HDO mechanism was proposed to involve several steps (Figure 35) beginning with the deprotonation of **1^OH^** to **1^O−^**, verified by NMR spectroscopy. The presence of a π-donor ligand enhances the reaction by increasing acidity. Subsequently, the binding of H_2_ to the active site leads to dihydride intermediates that can be deprotonated by a base. The remaining hydride thus attacks the benzylic -CH_2_ of VA to yield Product A, leaving a -OH group. Upon protonation, the -OH group becomes an aqua ligand and detaches to regenerate the catalyst. The mechanism suggests inner-sphere reactions, with coordination of VA to the metal center facilitating the formation of Product A at the benzylic position—a reactivity not observed with phenolic OH groups due to the difficult substitution of *sp*^2^ C–H [53].

Hydrosilylation is an important reaction producing a range of common chemical goods; yet this reaction still suffers from high catalyst loading, and so it is favorable to develop a bimetallic system with closely aligned metal active sites for synergistic activation of substrates. Pernik and co-workers carry out a comparison between monometallic and bimetallic Rh(I) complexes featuring NHC/phosphine bidentate ligands (**55a** and **55b**) using hydrosilylation of diphenylacetylene with triethylsilane as a model reaction for the study. NMR and ESI-MS data suggest a similar chemical environment for both monometallic and bimetallic Rh(I) analogues. In silane-premixed THF, the bimetallic catalyst achieved over 96% conversion within 1.5 min and a TOF of 2800 h^−1^—7.4 times higher than the monometallic version at 380 h^−1^ and over 100 times faster than previously reported bimetallic Rh-NHC/pyrazole catalysts. Notably, it selectively produced the *E*-isomer of the vinylsilane as the only product.

**Figure 36:**
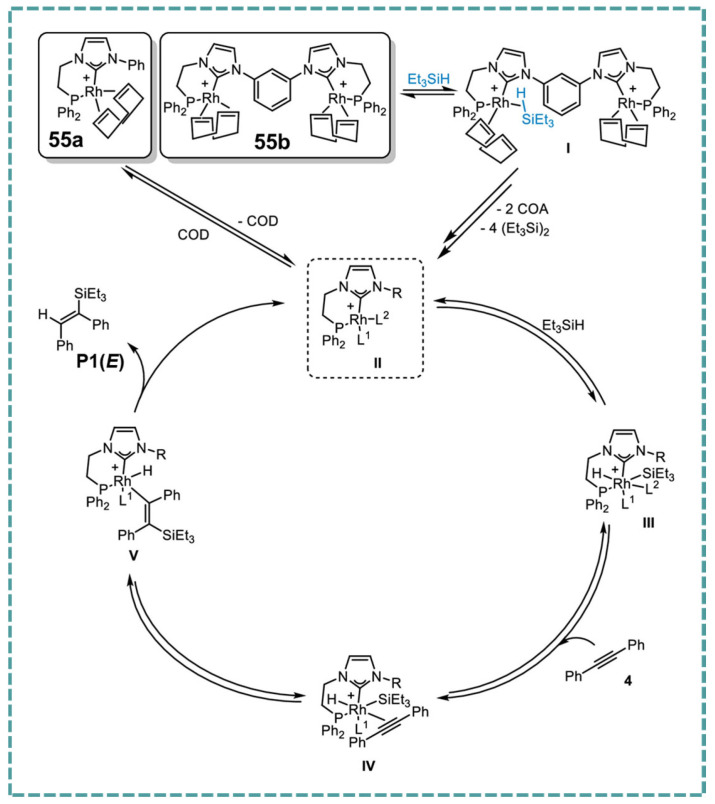
Proposed major hydrosilylation pathways for the monometallic and bimetallic catalysts **55a** and **55b**, depicted using **S1** as the substrate (L^1^ and L^2^ = solvent or **S1**).

Mechanistic studies revealed that bimetallic enhancement primarily arises from synergistic preactivation. The dinuclear structure promotes dynamic dissociation of cyclooctadiene (COD) ligands, facilitating their reduction to cyclooctane (COA) by silane and freeing active sites for the reaction, which follows the Chalk–Harrod mechanism where sequential Si-H bond activation, alkyne coordination, hydride migration, and reductive elimination occur to yield the final products (Figure 36). Density functional theory (DFT) calculations indicated that intramolecular hydrogen transfer lowers activation barriers, while ordering the substrate addition (silane-first) and utilizing polar solvents like THF optimized reaction pathways. This work uncovers a mechanism where ligand dynamics and spatial proximity improve preactivation efficiency in bimetallic catalysts, providing insight for rational design of high-performance industrial catalysts (e.g., silicone rubber synthesis) and future studies of heterobimetallic systems in photo- or electro-catalytic applications [31].

**Figure 37:**
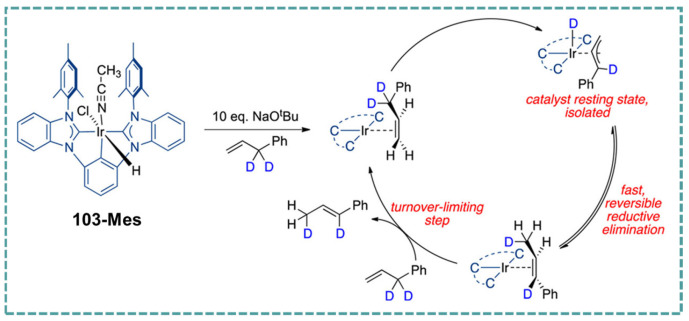
Proposed mechanism of catalytic alkene isomerization using **103-Mes** or **104-Mes**.

Chianese et al. investigated the mechanistic differences in alkene isomerization catalyzed by two CCC-pincer bis-NHC based Ir(III) complexes with either mesityl groups (**103-Mes**) or adamantyl groups (**103-Ad**). It was found that ligand steric effects greatly influenced the reaction pathways. For **103-Mes**, supported by ^13^C NMR results showing exclusive deuteration at C1 and C3 without intermolecular H/D crossover, the π-allyl mechanism was confirmed to primarily originate from intramolecular 1,3-deuterium migration in deuterated allylbiphenyl (*d*^2^-allylbiphenyl) (Figure 37). The η^3^-allyl hydride intermediate (**104-Mes**) was isolated and crystallographically resolved along with kinetic studies that determined a first-order rate constant (*k*) of 0.177 M^−1^ s^−1^. The exchange spectroscopy (EXSY) further showed rapid C-H reductive/oxidative elimination/addition between **104-Mes** and η^2^-alkene intermediates. In contrast, **103-Ad** exhibited a mixed mechanism, with predominant 1,3-deuterium shifts and intermolecular hydrogen transfer, suggesting either a selective insertion/elimination pathway via secondary metal-alkyl intermediates (anti-Markovnikov alkene insertion) or an unidentified hydrogen exchange pathway (e.g., base-mediated Ir-H/Ir-D exchange). Divergent mechanisms were attributed to the ligand steric effect of the bulky mesityl and adamantyl groups. Mesityl in **103-Mes** stabilized the π-allyl intermediate and suppressed ligand exchange, while the adamantyl in **103-Ad** promoted dynamic interactions and hydrogen transfer. Thus, **103-Mes** is better for precise synthesis (e.g., *E*-isomer production) while **103-Ad**, achieving a TON of 487 in 30 min for allylbenzene isomerization, holds potential for industrial applications. Overall, this study highlights how ligand engineering can modulate catalytic mechanisms in transition-metal systems, offering valuable insights for developing selective C-H functionalization catalysts [54].

### 4.4. Organic Light-Emitting Devices

**Figure 38:**
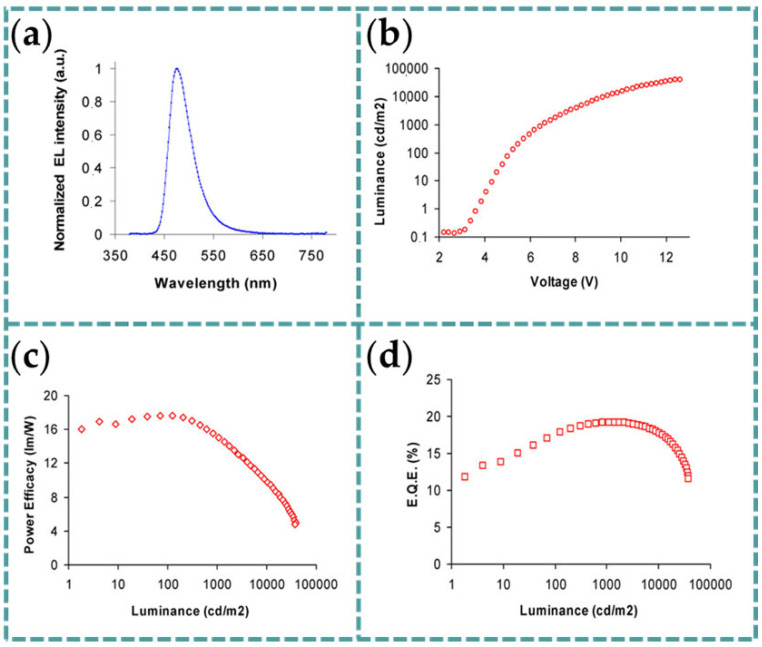
Plots of (**a**) electroluminescent spectra for the OLED device based on **44**, (**b**) luminance vs. voltage, (**c**) power efficiency vs. luminance, and (**d**) EQE vs. luminance.

OLEDs are emerging as the next generation for display and lighting uses, and phosphorescent emitter-based OLEDs (PHOLEDs) are especially attractive due to their ideal 100% quantum efficiency through thermally activated delayed fluorescence. Heavy-metal ions (e.g., Ir(III), Pt(II), Os(II), etc.) containing triplet emitters have been applied to develop PHOLEDs. Even though Os(II) has rather energetically shallow HOMOs as good hole traps, strong π-backbonding to the ligand, promoting MLCT transition for high photoluminescence quantum yields, and a short excited-state lifetime, reducing excited-state degradation or triple-triple annihilation, Os(II) blue emitters are rare, stemming from the challenging synthesis and energetically shallow HOMO. In 2014, Esteruelas and Xia reported Os(II) complexes featuring bis-tridentate NHC-based pincers as blue–green PHOLEDs. A series of homoleptic **[Os^II^(C_NHC_C_aryl_C_NHC_)_2_]** and heteroleptic **[Os^II^(C_NHC_C_aryl_C_NHC_)(C_NHC_C_aryl__’_C_NHC_)]** complexes were studied (Scheme 13). The homoleptic complex shows a phosphorescence quantum yield (Φ) of 0.62 in the solid state and an excited-state lifetime (τ) of 28 μs, effectively suppressing triplet–triplet annihilation (TTA). TD-DFT calculations indicate that its luminescence results from MLCT and ligand-centered π-π* transitions. The high HOMO energy level enhances hole trapping and facilitates carrier recombination. The electroluminescence spectrum of the OLED device peaks at 476 nm with CIE coordinates of (0.14, 0.26) and a maximum external quantum efficiency (EQE) of 19.2% at 1000 cd m^−2^ (Figure 38), setting a new benchmark for Os-based blue emitters. Additionally, solution processability was confirmed through vacuum deposition, achieving a luminance of 10,000 cd m^−2^ at 9.5 V, which demonstrates commercial viability. Nevertheless, challenges remain in developing deep-blue emission (CIE y < 0.2) and near-infrared materials, with the high cost of Os limiting large-scale applications [27].

**Figure 39:**
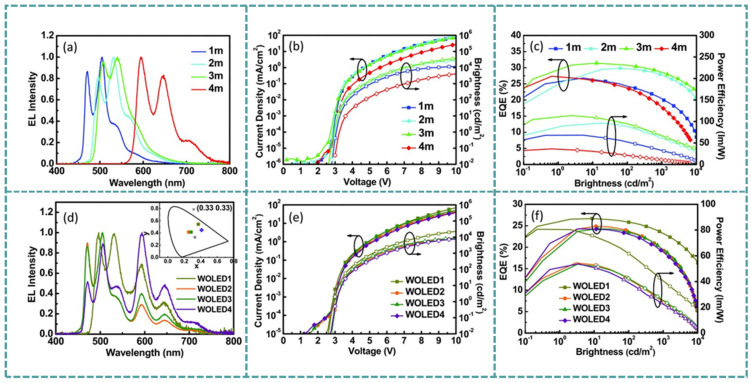
EL characteristics of OLEDs incorporating the studied emitters **69**, **70**, **71** and **75**: (**a**) EL spectra, (**b**) *I*-*V*-*L* characteristics, and (**c**) external quantum and power efficiencies, and EL characteristics of white OLEDs incorporating the studied emitters **70** + **75** (**WOLED1**) and **69** + **75** (**WOLED2**−**WOLED4**); (**d**) EL spectra recorded at the bias voltage of 6 V, corresponding to a luminance of 1000–2400 cd m^−2^, (**e**) *I*-*V*-*L* characteristics, and (**f**) external quantum and power efficiencies. The inset in panel (**d**) depicts the 1931 CIE coordinates of **WOLED1**–**WOLED4**.

In 2016, Chou et al. designed a series of neutral bis-tridentate Ir(III) complexes bearing phenylene-bridged bis-NHC pincers (Figure 16). These complexes achieve full-spectrum emissions ranging from blue to red with high efficiency through ligand rigidification and electronic structural modulation. The methyl-substituted complexes (e.g., **69**, **70**, **71**) demonstrate near-unity phosphorescence quantum yields (Φ = 99%) in solution, with excited-state lifetimes ranging from 4.6 to 8.2 μs. In contrast, the isopropyl-substituted analogues (e.g., **73**, **74**) further enhance the quantum yields to 97-98% in solid-state films due to steric hindrance effects. TD-DFT calculations indicate that their luminescence arises from a synergistic MLCT and IL. The optimal alignment of the HOMO energy levels improves charge-carrier injection efficiency with the bipolar host material 9-(3-(9*H*-carbazol-9-yl)phenyl)-9*H*-carbazole-3-carbonitrile, mCPCN. Furthermore, the isopropyl-substituted complexes exhibit horizontal dipole orientation ratios of 74-78%, which significantly enhances light outcoupling efficiency, allowing for high EQE in the OLED devices. The green OLED device, based on **71** with 12 wt% doping, achieves a maximum EQE of 31.4% and a power efficiency of 108.7 lm W^−1^, showing CIE coordinates of (0.34, 0.60) and an EQE of 28.4% at 1000 cd m^−2^ (Figure 39c). The red-emitting device, **75** with 1 wt% doping, delivers an EQE of 27.4% and CIE coordinates of (0.63, 0.38) (Figure 39a). White OLEDs like WOLED1 combine blue emitter **69** and red emitter **75**, achieving an EQE of 26.7% and a color rendering index (CRI) of **74**, demonstrating excellent color fidelity (Figure 39d,f). The architecture uses MoO_3_/LiF charge injection layers and the bipolar host mCPCN, allowing for low turn-on voltages of 2.5-2.9 V and high luminance of 10,000 cd m^−2^ (Figure 39b,e) [36].

**Figure 40:**
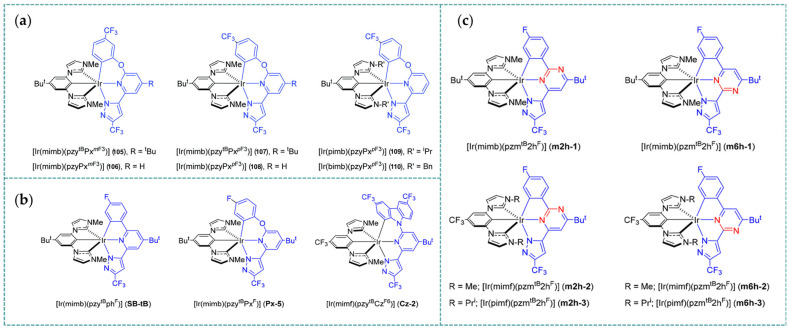
(**a**) Structures of Ir(III) complexes **Px-11**-**Px-16** with a functional phenoxy appendage; (**b**) sky-blue- and blue-emitting bis-tridentate Ir(III) phosphors **SB-tB**, **Px-5**, and **Cz-2**; (**c**) structures of bis-tridentate Ir(III) complexes **m2h-1**-**m2h-3** and **m6h-1**-**m6h-3** bearing a pyrimidinyl functionality.

Considering the development of Ir(III) bis-tridentate bis-NHC-based complexes, Chi and co-workers contributed several works, revealing their efficient luminescent mechanisms for applications in organic light-emitting diodes (OLEDs). Their research highlights the importance of ligand engineering in influencing photophysical properties and device performance, providing vital insights for the development of deep-blue OLEDs. In 2018, the team utilized 6-pyrazolyl-2-phenoxy-pyridine (**pzyPx**) ligands to prepare bis-tridentate Ir(III) complexes (**Px-11**-**16**) (Figure 40a), which show robust photoluminescence centered at 448 nm, with quantum efficiencies of almost 100% and an excited-state lifetime of ~10^1^ μs [47]. When adding CF_3_ and *tert*-butyl substituents (Figure 40b), these complexes achieved near-unity quantum efficiencies with reduced radiative lifetimes of 3.4–10.0 μs. Subsequent studies with pyrimidine-based ligands (Figure 40c) [55] improved MLCT pathways, resulting in a maximum EQE of 17.6% for green (515 nm) and 15.9% for sky-blue (473 nm) OLEDs, surpassing the traditional ***fac*-[Ir(ppy)_3_]**. In 2021, they designed phenylene-bridged imidazo [4,5-b]pyridine-based bis-NHC ligands [48], producing a deep-blue emitter **Dap-5** (CIE (0.14, 0.15)) with a radiative lifetime of 0.67 μs and an EQE of 16.8%, demonstrating strong operational stability. Studies on charge-state modulation [49] have shown that neutral complexes exhibit a larger energy gap between the ^3^MLCT/ππ* state and the ^3^MC/*dd* state, achieving 100% quantum efficiency and outpacing cationic derivatives. Chi’s team demonstrates a clear advancement from traditional ligand systems to more effective heterocyclic approaches, merging short radiative lifetimes and strong ligand-field strength in optimizing the performance of Ir(III) triplet emitters and their derived OLEDs.

**Figure 41:**
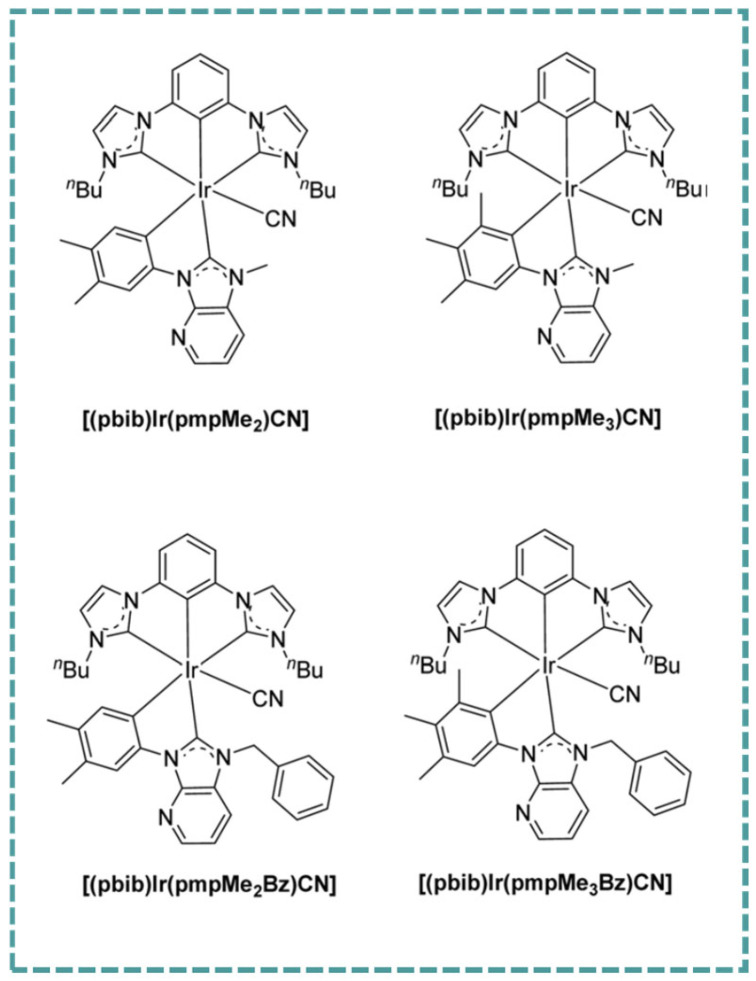
Deep-blue phosphorescent [3 + 2 + 1] coordinated Ir(III) complexes.

**Figure 42:**
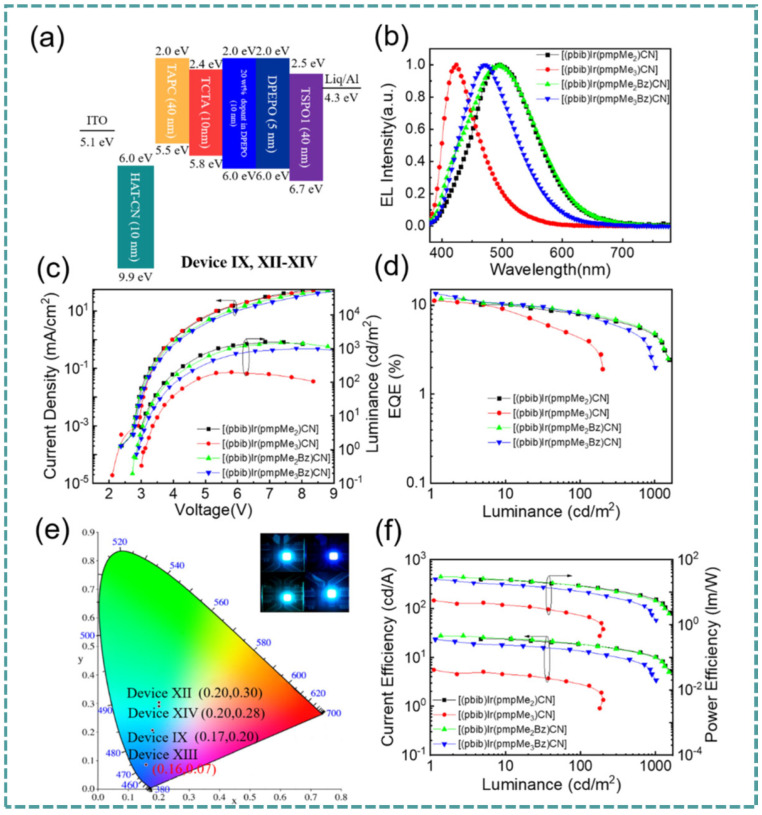
Devices IX and XII-XIV. (**a**) An energy diagram of the materials used in the OLEDs. (**b**) EL spectra of all devices. (**c**) Current density–voltage–luminance (*J*-*V*-*L*) characteristics. (**d**) EQE vs. luminance. (**e**) CIE coordinates. (**f**) Current efficiency–luminance–power efficiency (*CE*-*L*-*PE*).

To tackle efficiency roll-off and color purity issues in deep-blue OLED materials, Wei and Tang developed Ir(III) complexes with a [3 + 2 + 1] coordination geometry. These complexes feature a phenyl-3*H*-imidazo[4,5-*b*]pyridine (C^C) bidentate ligand, an NHC pincer ligand (pbib), and a -CN auxiliary ligand (Figure 41). By modifying substituents on the C^C ligand, the emission energy was finely tuned across 415-470 nm. The strong electron-withdrawing nature of the C^C ligand enhanced ILCT, shifting solution-state emission peaks to 418–424 nm and solid-state films to 426-434 nm, and achieving CIE coordinates of (0.16, 0.07), which are close to the European Broadcasting Union (EBU)’s standard of (0.15, 0.06). Theoretical calculations revealed that the excited-state spin density was primarily localized on the C^C ligand, thereby reducing nonradiative relaxation. Consequently, the solid-state photoluminescence quantum yield (PLQY) rose to 61%, with excited-state lifetimes of 0.3 to 1.1 μs and a high radiative decay rate (*k*_r_ = 10^6^ s^−1^), minimizing TTA and singlet-triplet annihilation (STA). The optimized device achieved a maximum EQE of 11.2% with a luminance of 1017 cd m^−2^ (Figure 42). This work provides a promising strategy for designing high-color-purity deep-blue phosphors, advancing next-generation ultra-high-definition display technologies [56].

Based on the efforts from different research groups, future research should prioritize several key areas to advance the development of high-efficiency phosphorescent materials based on NHC-based complexes. First, there is a need to incorporate strong electron-withdrawing groups, such as -CN, to facilitate blue emission shifts and develop deep-blue emitters (CIE y < 0.1) that can expand color gamuts. Second, exploring non-noble metal alternatives, particularly Cu and Zn-based complexes, presents an opportunity to reduce costs while enhancing performance. Additionally, optimizing solution-processable protocols is crucial for enabling the manufacturing of flexible, large-area OLED devices. To further enhance these directions, investigating exciton dynamics through ultrafast spectroscopy will be essential for elucidating the ratios of MLCT and LLCT. Ultimately, it aims to minimize the singlet-triplet energy gap (ΔE_ST_) and suppress efficiency roll-off, thereby improving overall device performance. Collectively, these structure–property correlation studies will not only deepen our understanding of bis-tridentate Ir(III) complexes but also lay experimental and theoretical foundations for the industrialization of efficient persistent blue OLEDs.

## 5. Conclusions and Perspectives

Since the isolation of stable NHCs by Arduengo in 1991, research on these carbon donors has spanned various fields and continues to thrive. Due to their strong σ-donating ability, pincer-type NHCs have been proposed as powerful alternatives to classical polypyridyl ligands for tuning the photophysics and photoluminescence of transition metal complexes. After three decades of development, pincer-type NHC-based complexes have found applications in various fields, including OLEDs, catalysis, and biological probes or medicines.

Despite these successes, the current research on these platforms remains limited to the molecular level, lacking modulation strategies for higher-dimensional properties such as multiple active sites, luminophores, topological tuning, and interspatial interactions. Given extensive research on pincer-type NHC-based coordination complexes, it is now time to elevate this category of complexes to the polymeric level. This transition would allow for the incorporation of supramolecular pore structures, network topology, on-demand multiple metal-centered active sites, and inter-linker interactions into the toolbox for application development.

Researchers have begun to extend NHCs to heterogeneous chemistry by immobilizing them on various material platforms [57,58,59,60]. Specifically, metal–organic frameworks (MOFs) and covalent–organic frameworks (COFs)—which are structurally well-defined crystalline polymers—serve as ideal platforms for integrating pincer-type NHC-based complexes as building blocks. This integration can facilitate connections with various units to open Pandora’s box of chemistry.

## Figures and Tables

**Table 1:** Catalyst performance data in the photocatalytic CO_2_ reduction system. Data is organized by decreasing TOF_max_.

Catalyst^[1]^	TON_max_	TOF_max_ [h^−1^]
**Ru^OMe^**	1006	174
**Ru^Me^**	639	104
**Ru^3OMe^**	565	32
**Ru^H^**	440	31
**Ru^H^** ^[2]^	120	14
**Ru^NPh2^**	337	13
**Ru^NMe2^**	38	4
**Ru^OH^**	15	1

^1^ All values come from duplicate experiments with a <5% difference in the two runs. ^2^ White solids formed during this reaction in multiple experiments, although the reaction was seemingly set up under identical conditions to the other entries.

## Data Availability

No extra data are included in this review article.
